# Investigating the evolutionary dynamics and mutational pattern of SARS-CoV-2 spike gene on selected SARS-CoV-2 variants

**DOI:** 10.1371/journal.pone.0333093

**Published:** 2025-10-21

**Authors:** Bachir Balech, Alessandra Lo Presti, Claudia Telegrafo, Lucia Maisto, Emanuela Giombini, Angela Di Martino, Luigina Ambrosio, Apollonia Tullo, Paola Stefanelli, on behalf of the Italian Genomic Laboratory Network

**Affiliations:** 1 Institute of Biomembranes, Bioenergetics and Molecular Biotechnologies, Consiglio Nazionale delle Ricerche, Bari, Italy; 2 Department of Infectious Diseases, Istituto Superiore di Sanità, Rome, Italy; Federal Medical Centre Abeokuta, NIGERIA

## Abstract

The continuous evolution of SARS-CoV-2 has led to the emergence of several variants representing significant challenges for public health. Many studies highlight the relevance of phylogenetic inference or mutational pattern analysis to understand the evolutionary relatedness of viral variants and to estimate the potential effect of new mutations on viral transmission, virulence and antigenicity. Here we describe an evolutionary investigation approach combined with mutational analyses of SARS-CoV-2 Spike gene to annotate and potentially track important amino acid site variation of specific functional domain relevant for viral survival. This approach was applied on XBB*, EG* and BA* and their sub-lineages (see materials and methods) available from GISAID. In addition, we considered the major variants of concern (Alpha, Delta, Omicron) and Wuhan-Hu-1 strain as references. Maximum likelihood phylogenetic tree was constructed from the complete dataset while selection pressure and mutational analyses were conducted on single variants separately. The obtained phylogenetic tree of Spike amino acid gene sequence showed a clear separation of viral variants as well as their expected appearance order. This result supported the significance of selection pressure analyses outcomes combined with amino acid mutational frequencies where in many cases they showed a linear and parallel trend. This allowed also to hypothesize the potential importance of low-frequency mutations in new potential virus variants. This study constitutes an asset of important insights to be considered in regular monitoring programs. In addition, the analysis framework described here introduces a starting point for further standardization, optimization and application on different data types and in large-scale studies.

## Introduction

The continuous evolution of the Severe Acute Respiratory Syndrome Coronavirus 2 (SARS-CoV-2) has led to the emergence of several variants, and distinct lineages and sub-lineages [1–3] representing significant challenges for public health and requiring a regular monitoring. XBB variant and its related sub-lineages replaced previously circulating Omicron variant in early 2023. XBB presents a strong capacity for crossing over the host immune system, surpassing the immune evasiveness of BA.5 [4]. The Omicron XBB.1.5 has become predominant in Italy starting from April 2023 [5]. It continued to evolve, yielding other main sub-lineages (i.e., XBB.1.9.1, XBB.1.9.2, XBB.1.16, XBB.2.3, FE.1) which circulated and played a key role in viral infection and transmission. In August 2023, this variant was replaced by the EG.5, which became the most prevalent in the country [6]. In the same period the lineage named BA.2.86, has been detected in multiple countries, prompting its classification as a Variant of Interest (VOI) by the World Health Organization (WHO) (World Health Organization 2024 [7]). According to data reported from the Italian flash survey report [8], this viral strain was detected in Italy for the first time in September 2023. The reproductive efficiency of BA.2.86 is estimated to be similar or even higher that of XBB.1.5 and EG.5.1 [9]. Although BA.2.86 did not show substantial humoral immune escape and growth advantage compared to EG.5 and EG.5.1 variants, it showed remarkably high ACE2 (angiotensin-converting enzyme 2) binding affinity [9–13]. Moreover, the defining and relevant mutations in the Spike of XBB.1.5*, XBB.1.9*, XBB.1.16*, XBB.2.3*, FE.1*, EG.5*, BA.2.86* described by the outbreak.info mutation tracker (reported and summarized in S1 Table), represent the main reasons to monitor these variants [14], along with the ability to monitor the onset of possible additional mutations which can arise in specific strains over time.

Spike protein is one of the structural proteins of SARS-CoV-2. It has a crucial role in fusion with the host cell, viral pathogenicity and vaccine design [15,16]. Diving into Spike mutational pattern has been the central focus of many scientific studies to date as it is related directly to the viral fitness [15,16]. The structure of the Spike protein comprises a signal peptide (SP, amino acids residues: 1−13) and two subunits the S1 (residues: 14−685) and S2 (residues: 686−1273) [17]. The S1 subunit contains an N terminal (NTD, residues:14–305) and a C-terminal receptor binding subdomains (RBD, residues: 319−541). The NTD has a critical role in overall structural conformation of S protein, where mutations occurring in the NTD are linked to viral immune escape [18]. The RBD instead is responsible for the recognition of the angiotensin-converting enzyme 2 (ACE2) which acts as the receptor for SARS-CoV-2 viral entry [19]. The S2 subunit comprises the fusion peptide (FP, 788–806 residues), heptapeptide repeat sequence 1 (HR1, 912–984 residues), heptapeptide repeat sequence 2 (HR2, 1163–1213 residues), transmembrane (TM) domain (1213–1237 residues), and cytoplasm domain or C-terminal tail (CT, 1237–1273) [20]. HR1 and HR2 lead to membrane fusion and viral entry, while the TM assures the anchoring of S to the viral envelop and the C-terminal tail promotes S escape from the endoplasmic reticulum [21]. Mutational changes associated with SARS-CoV-2 Spike protein coding gene have gained important insights to study the related evolutionary dynamics of the virus. Many studies highlight the relevance of complete genomes phylogeny of SARS-CoV-2 to illustrate its relatedness with other viruses of the same family namely, SARS-CoV and MERS [22–26]. In parallel, in some cases it has been demonstrated the importance of phylogenetic analysis of Spike gene as a potential region to discriminate among variants of the virus and at the same time a relatively fast method to flag or detect the outbreak of new variants and/or the appearance of new mutations or indels [27,28].

In this context, monitoring the evolution of SARS-CoV-2 variants and the emergence of specific amino acid substitutions or indels is determinant to detect potential alterations in transmissibility, infection severity, and immune responsiveness, and to inform risk assessment and early warning models. This can be achieved through conducting a phylogeny inference to highlight newly emerging clade/s or by studying site-specific selection pressure [29]. Generally, the amino acid changes that increase virus fitness are generally maintained by positive selection.

Hereby, we endorsed a bioinformatic analytical approach to highlight important aspects of Spike gene related to the selection pressure, phylogenetic analysis and evolutionary dynamics in selected SARS- CoV-2 variants and their sub-lineages (details are provided in materials and methods section and S2 Table), as a model. This allowed the evaluation of Spike gene evolution and its mutational pattern (substitutions and indels) as well as the sites subjected to positive and negative selection and their relationship with specific functional domains crucial in regular monitoring and treatments developing programs including vaccine design.

## Materials and Methods

### Source datasets

Three complete genome sequences datasets were downloaded from GISAID database (https://gisaid.org/) on 25 September 2023. A total of 3736 SARS-CoV-2 available genomes belonging to XBB.1.5, XBB.1.16, XBB.2.3, FE.1 (alias of XBB.1.18.1.1.1) and their sub-lineages in addition to XBB.1.9.1 and XBB.1.9.2 from Italy were downloaded. Lineage and sub-lineage assignment have been done with pangolin v.4.3.1 through GISAID. Spike gene identification and extraction were performed following the analysis process described in detail in the subsequent sections. Following the exclusion of low-quality sequences with more than 5% of ambiguous nucleotides (N) in the Spike (S) protein coding gene, a total of 3724 S sequences were retained to constitute the first dataset. The second dataset included 436 Italian SARS-CoV-2 S gene sequences belonging to EG.5 variant and its sub-lineages. The third contained global sequences of BA.2.86 lineage (sampled from 208 different countries) and the only BA.2.86.1 Italian genome which was available at the time of the dataset creation. The same Spike gene identification procedure and sequence quality check described above were also applied to the other two datasets. Details on the number of sequences of each lineage/sub-lineage are reported in S2 Table. For the sake of simplicity in the rest of this manuscript we will refer to the first Spike gene sequences dataset as XBB*, the second as EG* and the third as BA*.

### Construction of Spike gene datasets

Clinically relevant SARS-CoV-2 variants were mainly considered to build a reference sequence dataset to be used for Spike gene annotation of the above-described datasets. For that, representative viral complete genome sequences were explored and retrieved from COVID19 data portal hosted by the European Nucleotide Archive (available at [30] and described in [31]). The reference dataset included 202 sequences belonging to Alpha, Delta and Omicron variants and the original Wuhan-Hu-1 (NCBI Accession Number: NC_045512.2). Using a python script, the Spike gene nucleotide and the corresponding amino acid sequences were extracted from the reference genomes according to the gene name and annotation features provided in the flat files. Multiple amino acid reference sequence alignment was generated using muscle [32] and the corresponding Hidden Markov Model (HMM) multiple alignment profile was constructed by *hmmpress* algorithm (HMMer 3.3 package: http://hmmer.org/).

### Multiple sequence alignments and Phylogenetic analysis of Spike gene

Multiple sequence alignments (MSA) of the three Spike gene sequences datasets were conducted following the general schema of MSA-PAD 2.0 [33,34] with modified features accounting for the occurrence of unassigned nucleotides characters “N”. In details, all DNA sequences of the three datasets (XBB*, EG*, BA*) were translated into amino acids using the universal genetic code and all six open reading frames. Using *hmmsearch* algorithm (HMMer 3.3 package), the amino acid sequences were searched against the Spike gene reference HMM profile generated in the previous step to annotate the relevant Spike region in genome sequences. The extracted amino acid Spike gene sequences were then aligned by *hmmalign* (HMMer 3.3 package) against the Wuhan-Hu-1 Spike gene reference. Amino acid multiple sequence alignments were then back-aligned into nucleotide multiple alignments using ad hoc python script (for more details see [33,34]) and used for selection pressure analysis (see section 5.5 – selection pressure analysis). To draw the evolutionary relationship between the SARS-CoV-2 variants under investigation and their reference, a single amino acid MSA was constructed by joining all the datasets described above. This MSA included either the representative reference sequences of the past variants and those relative to the retrieved sequences used in this study. In addition, to account for the excessive computational capacity needed in phylogeny construction, the number of sequences in the MSA was reduced to a minimum set of 685 where each retained sequence contains at least one amino acid substitution site compared to Wuhan-Hu-1 reference. In such way all amino acid substitutions were represented in the final MSA subset at least once.

Phylogenetic relationship among variants was inferred using the Maximum Likelihood method and the HIVw+F+I+G4 evolutionary model as implemented in IQTREE package [35]. The best evolutionary model was selected among 168 amino acid sequence evolutionary models tested with model finder algorithm available from IQTREE. Node supports were obtained from non- parametric ultrafast bootstrap [36] analysis with 1000 replicates while branch lengths were optimized using the NNI algorithm. The consensus phylogenetic tree was edited using iTOL web service [37] where clades representing the same variants (except for XBB* and EG*) were collapsed manually to improve tree rendering and interpretation.

### Amino acid substitutions, indels and their frequencies

The SARS-CoV-2 reference sequence Wuhan-Hu-1 (NC_045512.2) was used as reference for all individual datasets, including previous variants, to call the amino acid substitutions, deletions and insertions (indels). Their frequencies (%) were calculated based on the number of sequences of the same variant/lineage. This was achieved using *ad hoc* python script including standardized functions able to extract the mutational pattern either from single or multiple variants.

### Selection pressure analysis

To investigate the SARS-CoV-2 positively and negatively selected sites, selection pressure analysis was performed separately on the Spike gene nucleotide MSAs generated previously for XBB*, EG* and BA*. A positive diversifying selection was inferred on sites statistically significant for a value of non- synonymous/synonymous substitution ω > 1, while negative selection was inferred for ω < 1 [38]. On the contrary, neutrality was inferred if ω = 1 [38].

The models **F**ast **U**nconstrained **B**ayesian **A**pp**R**oximation (FUBAR) [39,40], **F**ixed **E**ffects **L**ikelihood (FEL) [39], **S**ingle- **L**ikelihood **A**ncestor **C**ounting (SLAC) [39] and **M**ixed **E**ffects **M**odel of **E**volution (MEME) [41] of the HYPHY and data monkey softwares available under Galaxy platform [39] were used. A posterior probability ≥ 0.95 for FUBAR and p-value ≤ 0.1 for FEL, SLAC and MEME were used to infer significant selection. Only sites found under significant selection were reported. The positions of the sites under selection and the amino acid substitutions type in all the tested datasets were referred according to the SARS-CoV-2 reference sequence Wuhan-Hu-1 (NC_045512.2).

### Spike protein stability prediction

In-silico prediction of amino acid mutations detected under selection pressure was conducted using Site Directed Mutator [42] (SDM: https://compbio.medschl.cam.ac.uk/sdm2/). It is a statistical framework that calculates a stability score, analogous to the free energy difference (ΔΔG) between wild-type and mutant protein. Each amino acid mutation detected in the above datasets was tested singularly against the wild-type available from Protein Data Bank (PDB: https://www.rcsb.org/) database (accession number: 7cwn). ΔΔG positive or negative values represent an increased or decreased stability effect of each mutation respectively.

## Results

### Phylogenetic analyses

**Fig 1:**
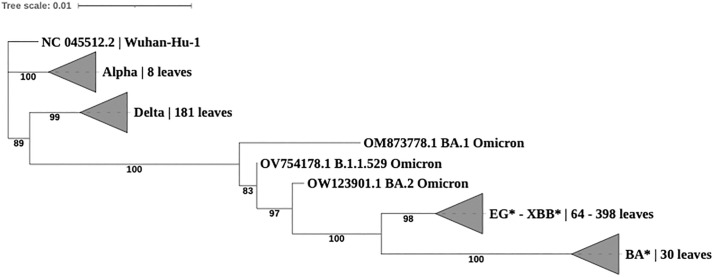
Maximum likelihood phylogenetic Tree of Spike protein gene obtained from IQTREE.

Phylogeny was inferred on Spike gene amino acid sequences to show the evolutionary relationship and genetic distance based only on non-synonymous mutations that occurred since the first appearance of Sars-CoV-2 until September 25^th^, 2023 (the date in which the present data was retrieved). Fig 1 shows a simplified phylogenetic tree collapsed per clades containing a single variant except for EG* and XBB* as they were found to share closer evolutionary distance within the same clade. All the other variants appeared in separated clades. The Wuhan-Hu-1 sequence was set as outgroup to infer the phylogenetic distance. This tree highlights the capacity of the Spike gene amino acid composition to discriminate almost all represented variants in our dataset with high statistical confidence shown from the bootstrap values. In addition, the tree shows the genetic distance as well as the appearance order of the different variants over time as follows: Alpha, Delta, Omicron, XBB*&EG* and BA*. The complete phylogenetic tree including all sub-lineages is available as S1 Fig.

### Spike amino acid substitutions and indels

Spike amino acid mutational pattern was identified taking the Wuhan-Hu-1 sequence as reference. A total of 662 substitutions (S3 Table) were detected across all analysed datasets. In details, Alpha variant showed the lowest number of substitutions (10) followed by Omicron (42), Delta (87), BA* (82), EG* (124) and XBB* (532). As reported in Table 1 and shown in S2 Fig, 91 substitutions were prioritized across all datasets showing a frequency higher than 10% in at least one of the analysed variants. Almost 85% (77 out of 91) of the high frequency mutations fall in the S1 subunit and distributed as follows: 29 in the NTD subdomain, 37 in RBD and 11 between S1 and S2. The mutations in S2 subunit are mainly localized in the HR1 subdomain (6) followed by two mutations in the fusion peptide and one in HR2. Importantly, the substitutions N501Y and D614G were detected in all variants, while A27S and G142D are shared across five variants (except Alpha). Following the outbreak of Delta variant, the mutational pattern of Spike gene appeared increasingly variable with additional accumulative substitutions as most of them are shared among Omicron, EG*, XBB* and BA* lineages and their corresponding sub-lineages.

**Table 1:** High frequency amino acid substitutions of Spike protein coding gene. Frequency higher than 10% in at least one variant (Alpha, Delta, Omicron, XBB*, EG*, BA*) were considered to prioritize the substitutions. Each a.a. replacement was attributed to the functional Spike subunits and the corresponding subdomain. Frequencies are reported in percentage scale.

Site	Substitution	Alpha	Delta	Omicron	XBB*	EG*	BA*	Subunits	Subdomains
19	T-I	NA	NA	100.0	99.6	95.87	79.33	S1	NTD
19	T-R	NA	100.0	NA	NA	NA	NA		
21	R-T	NA	1.1	NA	NA	NA	76.92		
27	A-S	NA	0.55	66.67	99.4	98.39	86.54		
50	S-L	NA	NA	NA	NA	NA	98.56		
52	Q-H	NA	NA	NA	0.03	78.44	NA		
67	A-V	NA	NA	33.33	0.11	0.46	NA		
73	T-I	12.5	NA	NA	NA	NA	NA		
83	V-A	NA	NA	NA	94.74	96.79	NA		
95	T-I	NA	39.27	33.33	0.03	NA	1.92		
127	V-F	NA	NA	NA	NA	NA	99.52		
142	G-D	NA	100.0	100.0	98.44	97.48	97.6		
146	H-Q	NA	NA	NA	93.93	94.72	NA		
156	E-G	NA	98.34	NA	NA	NA	NA		
157	F-S	NA	NA	NA	NA	NA	97.6		
158	R-G	NA	NA	NA	NA	NA	97.6		
183	Q-E	NA	NA	NA	98.42	96.79	NA		
211	N-I	NA	NA	33.33	NA	NA	NA		
212	L-I	NA	NA	NA	NA	NA	99.04		
212	L-V	NA	NA	33.33	NA	NA	NA		
213	V-E	NA	NA	NA	99.09	99.54	NA		
213	V-G	NA	NA	66.67	0.16	NA	100.0		
213	V-R	NA	NA	33.33	NA	NA	NA		
214	R-E	NA	NA	33.33	NA	NA	NA		
216	L-F	NA	NA	NA	0.05	NA	100.0		
222	A-V	NA	18.79	NA	0.05	NA	0.97		
245	H-N	NA	NA	NA	NA	NA	99.52		
252	G-V	NA	NA	NA	90.12	88.3	NA		
264	A-D	NA	NA	NA	NA	NA	96.15		
332	I-V	NA	NA	NA	NA	NA	97.6		RBD
339	G-D	NA	NA	100.0	0.24	NA	NA		
339	G-H	NA	NA	NA	98.31	97.94	97.12		
346	R-T	NA	NA	NA	98.39	97.48	NA		
356	K-T	NA	NA	NA	0.03	NA	93.75		
368	L-I	NA	NA	NA	94.69	91.74	NA		
371	S-F	NA	NA	33.33	95.92	92.66	94.23		
371	S-L	NA	NA	66.67	NA	NA	NA		
373	S-P	NA	NA	100.0	96.21	95.18	95.68		
375	S-F	NA	NA	100.0	95.65	95.18	95.19		
376	T-A	NA	NA	33.33	93.9	94.95	95.19		
403	R-K	NA	NA	NA	0.027	NA	69.71		
405	D-B	NA	NA	33.33	NA	NA	NA		
405	D-N	NA	NA	33.33	95.81	94.27	95.67		
408	R-S	NA	NA	33.33	89.98	85.55	88.46		
417	K-N	NA	0.55	100.0	89.85	87.61	90.87		
440	N-K	NA	NA	100.0	94.52	91.74	94.71		
445	V-H	NA	NA	NA	NA	NA	88.94		
445	V-P	NA	NA	NA	94.27	91.28	0.96		
446	G-S	NA	NA	33.33	94.39	91.28	89.9		
450	N-D	NA	NA	NA	NA	NA	88.47		
452	L-R	NA	100.0	NA	0.027	NA	NA		
452	L-W	NA	NA	NA	NA	NA	86.06		
456	F-L	NA	NA	NA	1.66	70.41	NA		
460	N-K	NA	NA	NA	71.91	71.79	90.38		
460	N-K	NA	NA	NA	71.9119	71.789	90.3846		
477	S-N	NA	NA	100.0	73.82	75.46	91.83		
478	T-K	NA	100.0	100.0	70.35	75.69	91.35		
481	N-K	NA	NA	NA	0.08	0.23	89.42		
484	E-A	NA	NA	100.0	96.56	95.64	1.44		
484	E-K	NA	NA	NA	NA	NA	84.13		
486	F-P	NA	NA	NA	96.88	97.25	93.75		
490	F-S	NA	NA	NA	97.13	98. 4	0.48		
493	Q-R	NA	NA	100.0	NA	NA	NA	S1	
496	G-S	NA	NA	33.33	NA	NA	NA		
498	Q-R	NA	NA	100.0	96.89	97.48	95.67		
501	N-Y	100.0	0.55	100.0	96.91	97.48	95.67		RBD
505	Y-H	NA	NA	100.0	96.72	97.02	97. 6		
547	T-K	NA	NA	33.33	NA	0.69	NA		
554	E-K	NA	NA	NA	0.13	NA	99.52		
570	A-D	100.0	NA	NA	NA	NA	NA		
570	A-V	NA	NA	NA	NA	NA	99.52		
583	E-D	12.5	0.55	NA	0.11	NA	NA		
614	D-G	100.0	100.0	100.0	99.41	98.85	100.0		
621	P-S	NA	NA	NA	0.11	NA	100.0		
655	H-Y	NA	NA	100.0	99.76	99.78	99.52		
679	N-K	NA	NA	100.0	99.57	97.94	99.52		
681	P-H	100.0	NA	100.0	99.78	98.85	NA		
681	P-R	NA	100.0	NA	NA	NA	99.52		
716	T-I	100.0	NA	NA	0.08	NA	NA		
764	N-K	NA	NA	100.0	99.06	98.85	99.04		
796	D-Y	NA	0.55	100.0	99.49	99.77	96.63		FP
797	F-L	NA	NA	33.33	NA	NA	NA		
856	N-K	NA	NA	33.33	NA	NA	NA		
939	S-F	NA	NA	NA	0.11	NA	99.52		
950	D-N	NA	100.0	NA	NA	NA	NA	S2	
954	Q-H	NA	NA	100.0	99.92	100.0	99.52		
969	N-K	NA	NA	100.0	99.81	99.09	100.0		
981	L-F	NA	NA	33.33	NA	NA	NA		
982	S-A	100.0	NA	NA	NA	NA	NA		HR1
1118	D-H	100.0	NA	NA	NA	NA	NA		
1143	P-L	NA	NA	NA	0.05	NA	100.0		
1191	K-N	12.5	0.55	NA	NA	NA	NA		HR2

Blue: Shared by 6 variants. Green: Shared by 5 variants. Red: Shared by 4 variants.

NTD: N-terminal subdomain (14-305 residues). RBD: C-terminal receptor binding subdomain (319-541 residues). FP: fusion peptide (FP) (788-806). HR1: heptapeptide repeat sequence 1 (912-984 residues), HR2: Heptapeptide repeat sequence 2 (1163-1213 residues).

**Fig 2:**
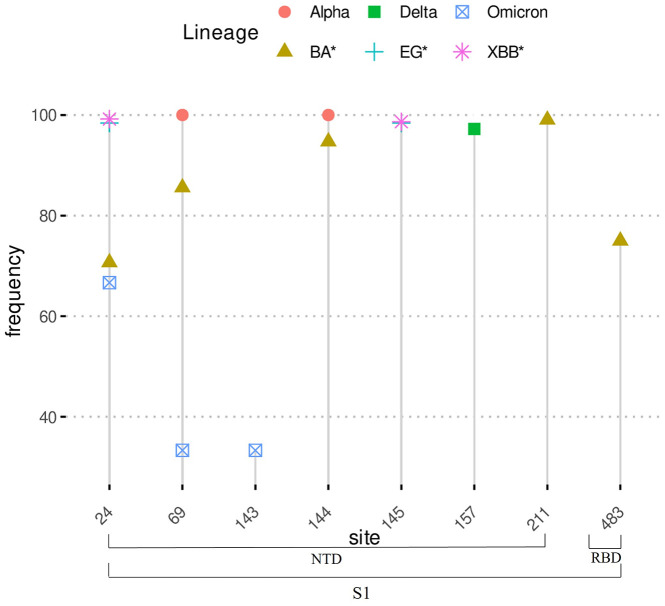
High frequency Spike gene amino acid deletions sites.

Beside the amino acid substitutions, the detected deletions and insertions were less frequent. A total of 33 deletions were found (S4 Table), where most of them appeared to be specific for a single variant. Fig 2 shows a pattern of shared and specific deletions with high frequencies across all datasets. The most frequent (>10%) deletions (n = 8) occurring in at least one of the datasets fall only in the S1 subunit and mainly in the NTD subdomain (Fig 2). Some deletions, such as ‘LPP’ at site 24 appear to be conserved in Omicron and its descendants (XBB*, EG*, BA*), while ‘HV’ at site 69 is shared among Alpha, Omicron and its related sub-lineages XBB* and BA*. Tow deletions are specific to Delta (at sites 156 and 157) and one to Omicron at site 143. At site 144 the deletion ‘Y’ with high frequency in Alpha seemed to be lost in Delta and Omicron and appeared again more recently in XBB* and BA* sub-lineages. The remaining deletions seem to have a recent outbreak mainly in XBB*, EG* and BA*.

Although the number of identified insertions appeared relatively low in all analysed datasets, their importance in viral evolution, survival and interaction with the host remains critical [43]. In total, three insertions within NTD S1 subdomain were detected with frequencies less than 50%, each belonging to single variant or sub-lineage (Table 2). The insertion ‘MPLF’ at site 17 was found in BA* dataset (45.7%), followed by ‘PE’ at site 215 (33.3%) in Omicron and ‘SLG’ in EG* (0.23%) at site 186.

**Table 2:** Insertions detected in Spike gene amino acid sequences with their corresponding positions and frequencies. All insertions belong to NTD functional subdomain of S1 subunit.

Site	Insertion	Omicron-Frequency	EG-Frequency	BA-Frequency	Subunits	Subdomains
17	MPLF	NA	NA	45.67		
186	SLG	NA	0.23	NA	S1	NTD
215	PE	33.33	NA	NA		

### Selection pressure, a.a. frequencies, protein stability and host-immune response

The selection pressure analysis performed with HYPHY software evaluated the presence of diversifying and purifying selection in the three Spike protein coding gene datasets. A total of 122, 30 and 9 sites predicted under positive selection pressure are reported in Table 3 a, b, c for XBB*, EG* and BA* datasets respectively.

**Table 3:** Selection pressure results obtained from spike protein coding gene with HYPHY software of a) XBB*, b) EG* and c) BA* datasets. The frequencies of amino acids mutation found under selection pressure are reported in the same order and separated by semi-colon. The predicted ΔΔG and the resulting effect on protein stability (+ for increased, – for decreased) are given for each corresponding mutation. The green colour indicates the sites involved in binding affinity to ACE2 (BAA) or Immune Escape (IE) according to deep-mutational scanning studies. The conserved mutation in the variant of interest, which appeared subsequently, according outbreak.info (namely JN.1 and its 949 sub-lineages) are signed with an asterisk (*).

a) XBB*								
Site	a.a. reference	a.a. identified in XBB*	Methods	Frequency	Predicted ΔΔG	Stability	Effect	Subdomain
Positively selected sites (ω for sites > 1)								
5	L	(L; F)	MEME	1.02				SP
25	P	(P; Y)	FUBAR/MEME	0.21	−0.6	–		NTD
80	D	(D; E; N; Y)	FUBAR	0.03; 0.03; 0.08	0.47; 0.29; −0.9	+; + ; -	IE^2^	
83	V	(V; A)	SLAC/FUBAR/MEME	94.74	0.33	+	IE^1^	
85	P	(P; Y)	MEME	0.03	2.52	+		
87	N	(N; A)	MEME	0.03	1.16	+		
88	D	(D; S)	MEME	0.02	−0.43	–		
89	G	(G; T)	MEME	0.03	−1.85	–		
91	Y	(Y; V)	MEME	0.03	−0.5	–		
93	A	(A; S)	MEME	0.21	−1.89	–		
137	N	(N; M)	MEME	0.03	0.25	+		
138	D	(D; Q)	MEME	0.05	−1	–		
139	P	(P; V; L)	MEME	0.03; 0.03	0.32; 0.37	+; +		
142	G	(G; D*)	SLAC/FUBAR/MEME	98.44	−1.98	–	IE^2^	
145	Y	(Y; D; Q; H)	MEME	0.05; 0.05; 0.03	−1.04; −0.78; −1.04	-; -; -	IE^2^	
146	H	(H; P; Q; K)	SLAC/FUBAR/MEME	5.21; 0.11; 93.93	−0.5; −1.23; −0.38	-; -; -		
148	N	(N; K; T)	FUBAR/MEME	0.03; 0.16	0.19; 0.1	+; +		
180	E	(E; K; Q; V)	MEME	0.03; 0.05; 2.98	−0.33; −0.06; 0.95	-; -; +		
182	K	(K; I; N; Q; R)	FUBAR/MEME	0.03; 0.48; 0.29; 0.03	−0.01; 0.3; 0.58; na	-; + ; + ; na		
183	Q	(Q; E; D)	FUBAR/MEME	98.42; 0.03	−0.18; −0.57	-; -	IE^1^	
184	G	(G; V)	MEME	0.29	−2.97			
222	A	(A; V; T; S)	MEME	0.05; 0.03; 0.11	0.86; −0.22; −0.59	-; -; -	IE^2^	
244	L	(L; F)	MEME	0.03	−0.78	–		
252	G	(G; A; S; V)	SLAC/FUBAR/MEME	0.02; 0.05; 90.12				
253	D	(D; G; Y)	FUBAR/MEME	2.44; 0.03			IE^2^	
257	G	(G; D; S)	MEME	0.03; 0.16	−0.86; −0.41	-; -	IE^2^	
323	T	(T; I)	SLAC/FUBAR/MEME	1.13	0.4	+		RBD
339	G	(G; D; H*; N; Y)	SLAC/FUBAR/MEME	0.24; 93.31; 0.03; 0.03	0.17; 0.38; −0.17; 0.93	+; + ; -; +	IE^2^	
346	R	(R; I; S; T)	SLAC/FUBAR/MEME	0.05; 0.09; 98.39	0.79; −0.8; −0.46	+; -; -	IE^2^	
352	A	(A; S; V)	FUBAR/MEME	0.05; 0.11	−1.17; 0.19	-; +		
368	L	(L; I)	SLAC/FUBAR/MEME	94.68	−0.45	–		
371	S	(S; F*)	MEME	95.92	0.54	+	IE^2^	
375	S	(S; F*)	FUBAR/MEME	95.65	0.52	+	IE^2^	
376*	T	(T; A)	SLAC/FUBAR/MEME	93.9	−0.5	–	IE^1^	
395	V	(V; E)	MEME	0.03	−3.48	–	IE^1^	
405	D	(D; N*)	FUBAR/MEME	95.81	0.66	+	IE^1,2^	
408	R	(R; G; S*)	SLAC/FUBAR/MEME	0.16; 89.98	−1.07; na	-; na	IE^2^	
417	K	(K; N*)	SLAC/FUBAR	89.85	−1.34	–	IE^2^	
440	N	(N; K*)	FUBAR/MEME	94.52	−0.16	–	BAA/IE^1,2^	
444	K	(K; R)	MEME	1.21	0.57	+	IE^1,2^	
445	V	(V; P; S)	SLAC/FUBAR/MEME	94.23; 0.13	0.6; −0.58	+; -	IE^2^	
446	G	(G; I; S*)	FUBAR/MEME	0.05; 94.39	−2.74; −4.11	-; -	BAA/IE^1,2,3^	
456	F	(F; L)	SLAC/FUBAR/MEME	1.66	−0.2	–	BAA/IE	
460	N	(N; K*)	SLAC/FUBAR/MEME	71.91	0.56	+	BAA/IE^1,2^	
477	S	(S; N*; D)	FUBAR/MEME	73.82; 0.03	0.22; 0.43	+; +	BAA/IE^1^	
478	T	(T; E; K*; Q; R)	SLAC/FUBAR/MEME	0.03; 70.35; 0.03; 3.6	−0.12; −0.24; −0.06; 0.08	-; -; -; +	IE^2^	
486	F	(F; H; L; P*; S; V)	SLAC/FUBAR/MEME	0.03; 0.05; 96.88; 0.03; 0.05	−0.19; −0.2; −1.7; −0.63; 0.61	-; -; -; -; +	BAA/IE^1,2^	
494	S	(S; P)	SLAC/FUBAR/MEME	0.32	−1.01	–	IE^2^	
505	Y	(Y; H*)	MEME	96.72	−0.06	–	BAA/IE^1,2^	
521	P	(P; S; T)	SLAC/FUBAR/MEME	2.68; 0.19	−0.63; −0.82	-; -		
554	E	(E; D; A; K*; Q)	FUBAR/MEME	0.08; 0.35; 0.13; 0.11	−1.32; −0.59; −0.39; −0.54	-; -; -; -		
583	E	(E; D; Q)	FUBAR/MEME	0.11; 0.11	−0.66; −0.1	-; -		
613	Q	(Q; H)	MEME	0.24	0.67	+		
657	N	(N; T)	MEME	0.21	0.04	+		
666	I	(I; V)	MEME	1.02	−2.15	–		
675	Q	(Q; H; K; R)	FUBAR/MEME	1.07; 0.05; 0.05	0.25; −0.04; 0.22	+; -; +		
679	N	(N; K*; M)	MEME	99.57; 0.03				
688	A	(A; V)	FUBAR/MEME	0.51				
764	N	(N; I; K*)	SLAC/FUBAR/MEME	0.03; 99.06	0.86; 0.7	+; +	IE^2^	
796	D	(D; Y*; H)	MEME	99.49; 0.11	0.87; 0.71	+; +	IE^1^	FP
834	I	(I; T; V)	MEME	0.03; 0.05				
883	T	(T; I)	MEME	0.91	1.51	+		
952	V	(V; I)	MEME	0.21	0.35	+		HR1
964	K	(K; E)	MEME	1.88	0.87	+		
978	N	(N; S)	MEME	0.27	0.18	+		
1045	K	(K; R)	MEME	1.1	0.27	+		
1117	T	(T; I)	SLAC/FUBAR/MEME	0.24	0.07	+		
1122	V	(V; A; L; M)	MEME	0.03; 0.03; 0.08	−172; −0.77; −1.16	-; -; -		
1124	G	(G; V)	SLAC/FUBAR/MEME	0.24	−1.72	–		
1128	V	(V; A; L)	FUBAR	0.05; 0.08	0.12; 0.47	+		
Negatively selected sites (ω for sites < 1)								
28	Y	(Y; H)	SLAC	0.03	−0.78	–		NTD
43	F	F	SLAC/FUBAR					
60	S	S	SLAC/FUBAR					
68	I	I	SLAC/FUBAR					
84	L	L	SLAC/FUBAR					
91	Y	Y	FEL					
99	N	N	SLAC					
104	W	W	FEL					
106	F	F	SLAC/FUBAR					
130	V	V	SLAC/FUBAR					
144	Y	(Y; L)	SLAC/FUBAR	0.03	−0.2	–		
234	N	N	FEL					
243	A	A	FUBAR					
245	H	(H; Y)	SLAC/FUBAR	0.13	0.29	+		
296	L	L	SLAC/FUBAR					
351	Y	Y	SLAC/FUBAR					RBD
354	N	(N; K)	SLAC	0.03				
410	I	I	SLAC/FUBAR					
411	A	A	FUBAR					
423	Y	Y	SLAC/FUBAR					
436	W	W	FEL					
439	N	N	SLAC/FUBAR					
441	L	L	FUBAR					
461	L	L	SLAC/FUBAR					
472	I	I	SLAC/FUBAR					
489	Y	Y	SLAC/FUBAR					
578	D	D	SLAC/FUBAR					
616	N	N	SLAC/FUBAR					
624	I	(I; M)	SLAC/FUBAR	0.03				
633	W	W	FEL					
691	S	(S; F)	SLAC/FUBAR	0.03	0.64	+		
709	N	N	SLAC/FUBAR					
740	M	M	FEL					
839	D	D	SLAC/FUBAR					
886	W	W	FEL					
900	M	M	FEL					
902	M	M	FEL					
935	Q	Q	SLAC/FUBAR					HR1
1029	M	M	FEL					
1046	G	G	SLAC/FUBAR					
1056	A	A	SLAC/FUBAR					
1060	V	V	FUBAR					
1067	Y	Y	SLAC/FUBAR					
1139	D	(D; H)	SLAC/FUBAR	0.03				
1171	G	G	SLAC/FUBAR					HR2
1178	N	N	SLAC/FUBAR					
1198	I	I	SLAC					
1212	W	W	FEL					
1214	W	W	FEL					TM
1215	Y	Y	SLAC/FUBAR					
1217	W	W	FEL					
1222	A	A	FUBAR					
1229	M	M	FEL					
1259	D	D	SLAC					CT
1261	S	S	SLAC/FUBAR					
1273	T	T	FUBAR					
b) EG*								
**Site**	**a.a. reference**	a.a. identified in EG*	**Methods**	**Frequency**	**Predicted ΔΔG**	**Stability**	**Effect**	**Subdomains**
Positively selected sites (ω for sites > 1)								
19	T	(T; I*; L)	FUBAR	95.87; 2.29	0.07; 0.5	+; +	IE^2^	NTD
52	Q	(Q; H)	FUBAR	78.44	0.22	+		
83	V	(V; A)	FEL/FUBAR/MEME	96.79	0.33	+		
142	G	(G; D*)	FUBAR	97.48	−1.98	–	IE^2^	
157	F	(F; L)	MEME	4.82	0.82	+		
183	Q	(Q; E; Z)	FUBAR/MEME	96.78; 0.69	−0.18; na	-; na		
184	G	(G; S)	MEME	2.52	−2.97	–		
252	G	(G; V)	FUBAR	88.3				
339	G	(G; H*)	MEME	97.97	0.38	+		RBD
368	L	(L; I; J)	FUBAR	91.74; 0.23	−0.45; na	-; na		
371	S	(S; F*)	FUBAR	92.66	0.54	+	IE^2^	
375	S	(S; F*; A)	MEME	95.18; 0.23	0.52; na	+; na	IE^2^	
405	D	(D; N*)	FUBAR	94.27	0.66	+	IE^2^	
408	R	(R; S*)	FEL/SLAC/FUBAR/MEME	85.55	−1.07	–	IE^2^	
417	K	(K; N*)	FUBAR	87.61	−1.34	–	IE^1,2^	
445	V	(V; P)	MEME	91.28	0.6	+	IE^2^	
455	L	(L; F)	FUBAR/MEME	0.917	−0.09	–	IE^2,3^	
456	F	(F; L)	FEL/SLAC/FUBAR/MEME	70.41	−0.2	–	IE^1,2^	
477	S	(S; N*)	FUBAR/MEME	75.46	0.22	+	IE^2^	
478	T	(T; K*; R)	FUBAR	75.68; 0.46	−0.24; 0.08	-; +	BAA/IE^1,2^	
486	F	(F; P*)	MEME	97.25	−1.7	–	BAA/IE^1,2^	
505	Y	(Y; H*)	FUBAR	97.02	−0.06	–	IE^2^	
704	S	(S; L)	MEME	2.75	0.77	+		
1122	V	(V; L)	MEME	0.98	−0,77	–		
1128	V	(V; L)	MEME	0.69	0.47	+		
Negatively selected sites (ω for sites < 1								
20	T	T	FEL/SLAC/FUBAR					NTD
41	K	K	FEL/SLAC/FUBAR					
101	I	I	FEL/SLAC/FUBAR					
109	T	T	FEL/SLAC/FUBAR					
144	Y	Y	FEL/SLAC/FUBAR					
201	F	F	FEL/SLAC/FUBAR					
295	P	P	FEL					
306	F	F	FEL/SLAC/FUBAR					
336	C	C	FEL/SLAC/FUBAR					RBD
343	N	N	FEL/SLAC/FUBAR					
364	D	D	FEL					
374	F	F	FEL					
384	P	P	FEL					
452	L	L	FEL/FUBAR					
699	L	L	FEL/FUBAR					
1140	P	P	FEL					
1146	D	D	FEL/SLAC/FUBAR					
c) BA*								
**Site**	**a.a. reference**	a.a. identified in BA*	**Methods**	**Frequency**	**Predicted ΔΔG**	**Stability**	**Effect**	**Subdomains**
Positively selected sites (ω for sites > 1)								
21	R	(R; T)	FUBAR	76.92	−0.17	–		NTD
95	T	(T; I)	MEME	1.92	0.07	+	IE^2^	
299	T	(T; I)	MEME	3.365	0.61	+		
339	G	(G; H*; Y)	FUBAR	97.11; 0.48	0.38; 0.93	+; +		RBD
403	R	(R; K*)	FUBAR	69.71			BAA^3^	
455	L	(L; S*)	MEME	1.44	−0.57	–	IE^2,3^	
477	S	(S; N*; D)	MEME	91.83; 0.96	0.22; 0.43	+; +	IE^2^	
922	L	(L; F)	MEME	1.92	−0.63	–		HR1
Negatively selected sites (ω for sites < 1) HYPHY software								
16	V	V	FEL/SLAC/FUBAR					NTD
20	T	T	FEL/SLAC/FUBAR					
668	A	A	FUBAR					
842	G	G	FEL/FUBAR					
899	A	A	FEL					
919	N	N	FEL/SLAC/FUBAR					HR1
1146	D	D	FEL					
1215	Y	Y	FEL					TM
1222	A	A	FEL					

**IE **= immune escape; **BAA **= binding affinity to ACE2; * = mutations present in JN.1* and sub-lineages; **SP = **signal peptide (residues: 1–13); **NTD **= N-terminal subdomain (14–305 residues); **RBD **=  C-terminal receptor binding subdomain (319–541 residues); **FP **= fusion peptide (788–806 residues); **HR1 **=heptapeptide repeat sequence 1 (912–984 residues); **HR2 =** heptapeptide repeat sequence 2 (1163–1213 residues); **TM **=** **transmembrane domain (1213–1237 residues); **CT **= C-terminal tail (1237–1273 residues).

Green = mined from deep-mutational scanning studies; ^1^ = [44] https://sars2.cvr.gla.ac.uk/cog-uk/; ^2^ = [45] https://pgx.zju.edu.cn/covepiab/; ^3^ = [46].

The XBB* dataset (Table 3a) revealed 71 significant positively selected sites, where 24 (24/71, 33.8%) are located in the RBD region (a. a. residues 319–541 according to [17]). 20 of the identified sites (20/71, 28.17%) were confirmed by three methods (SLAC/FUBAR/MEME) and 15 (21.13%) by two (FUBAR/MEME or SLAC/FUBAR). The amino acid replacements under positive selection appeared at different frequency intervals. In particular, 18% (20/122) were identified at frequency ≥ 90% (Table 3a) and 2.5% (3/122) between 70% and 74%. The remaining mutations showed frequencies between 0.023% and 5.2%. 56 were the significant negatively selected sites where 31 (55.3%) were confirmed by two methods (SLAC and FUBAR).

As shown in Table 3b, 25 positively selected sites were found in EG* dataset. 12% (3/25) were confirmed by at least three methods (sites 408 and 456 by FEL/SLAC/FUBAR/MEME and site 83 by FEL/FUBAR/MEME). 14 positively selected sites (14/25, 56%) are located inside the RBD, eight (32%) in NTD and three between S1/S2. These correspond to 30 amino acid substitutions, where 40% (12/30) were found at frequency ≥ 90% and 23,3% (7/30) between 70.4% and 88.3%. The remaining 11 mutations (11/30, 36.7%) appeared less frequent with values ranging between 0.23 and 4.8%. A total of 17 significant negatively selected sites were identified in this dataset, where 10 (58.8%) were confirmed by three methods (FEL/SLAC/FUBAR).

The BA* dataset indicated eight positively selected sites (Table 3c) where three were detected by FUBAR and five by MEME method. Four sites are located within RBD, three in NTD and one in HR1. In the positively selected sites of the BA* dataset 10 amino acid substitutions were identified. Two of which were detected at high frequency (≥ 90%), two (R21T, R403K) at frequencies of 77% and 70% respectively and the remaining between 0.5% and 3.4%. Evidence of supported negative selection was detected for nine sites, where four (44.4%) were reported by at least two methods.

The effect of the amino acid mutations under selection pressure on protein stability inferred from the Site-Directed Mutator (SDM) Model is also reported in Table 3 (a, b, c). Although most of the positively selected amino acids appear to confer a decreased stability to the Spike protein mainly in RBD region (i.e., in XBB*), many replacements refer to an opposite trend (mostly in BA*). However, almost all the reported values are not significantly distant from the baseline (zero), which strongly depends on the conformation of the crystalized protein 3D structure used to conduct the underneath simulations.

According to deep-mutational scanning data, most of the mutations under positive selection appear to have an Immune-Escape (IE) effect (30 sites in XBB*, 14 in EG*, 3 in BA*), while the others influence the binding affinity to ACE2 (7 sites in XBB*, 2 in EG*, 1 in BA*).

## Discussion

The SARS-CoV-2 virus has evolved rapidly since its first appearance leading to the emergence of several variants. Its genome is characterized by a certain level of diversity and complexity, driven by an accelerated evolutionary rate, resulting in the onset of new mutations and variants over time [14,47] which were classified as VOCs or VOIs by WHO [7]. These concepts highlight the importance of investigating the evolutionary dynamic of SARS-CoV-2, to better decipher the genetic variations and the key mutations under selection pressure that may have an impact on diagnosis, effective treatment and vaccine development. Furthermore, to keep up with the rapid evolution of the virus, it is important to combine different expertise, methodologies and bioinformatic frameworks to monitor and quickly identify the emerging changes (i.e., non-synonymous mutations) and their prevalence over a short period of time or in certain geographical area.

This study illustrates a potential approach to assess the selection pressure, the amino acid mutational pattern (substitutions and indels) and the evolutionary dynamics of the Spike protein coding gene of selected SARS-CoV-2 lineages and sub lineages (XBB*, EG*, BA* described materials and methods section and in S2 Table). Accordingly, the evolutionary relationship through phylogenetic analysis was investigated between the current (until September 25th, 2023, date of data retrieval) and the previous SARS-CoV-2 variants (Wuhan-Hu-1 strain, Alpha, Delta and Omicron). Although many studies illustrated the importance of phylogenetic reconstruction based on complete genome sequence information [22–26], here we explored the capacity of Spike gene alone, selecting *a priori* all the amino acid substitutions and indels, to draw the evolutionary relationship of SARS-CoV-2 variants since its first appearance in Wuhan. Despite the poor evolutionary resolution shown between XBB* and EG* derived sequences as they were placed in the same clade, Spike amino acid sequences alone provided a good tree topology showing a clear discrimination among the tested variants as well as their relative genetic distances (Fig 1). Based on these results, this well-known phylogenetic approach when applied on suitable genes (in our case Spike gene) or additional genomic region would represent a useful and accurate tool in monitoring programs of newly emergent variants without the need of the complete genome sequences. However, the complete genome information remains fundamental wherever the correct evolutionary resolution cannot be reached with single or multiple genes or a novel emerging pathogen is being analyzed.

As shown in Table 3, selection pressure analysis conducted in this study revealed an interesting pattern which was enriched by mutational frequency, deep mutational scanning data (mined from the litterature) and protein stability simulation for advanced Spike protein coding gene behavioural interpretation. For instance, 20 mutations identified as positively selected sites, were common between the XBB* and EG* (V83A, G142D, Q183E, G252V, G339H, L368I, S371F, S375F, D405N, R408S, K417N, V445P, F456L, S477N, T478K, T478R, F486P, Y505H, V1122L, V1128L) and four between the XBB* and BA* (G339H, G339Y, S477N, S477D). Many of these mutations (11 shared across all datasets) were found also in JN.1* and sub-lineages, the variant of interest appeared right after the sampling conducted in this study. This may suggest the possibility to identify potential “marker sites” expressed as conserved mutation regardless the specific lineage to which they belong. Additional investigation on these sites would provide important implications for targeted vaccine formulations and viral monitoring programs.

In XBB* dataset, among the positively selected sites with a.a. replacements observed at high frequency (≥ 90%), five (V83A, G142D, H146K, Q183E, G252V) were in NTD, 12 (G339H, R346T, L368I, S371F, S375F, T376A, D405N, N440K, V445P, G446S, F486P, Y505H) in RBD and one in the S2 subunit (D796Y in Fusion Peptide). In EG* dataset four mutations (T19I, V83A, G142D, Q183E) were in NTD and eight in RBD (G339H, L368I, S371F, S375F, D405N, V445P, F486P, Y505H). In BA* dataset, only two a.a. replacement (positively selected sites at frequency ≥ 90%) were detected in RBD (G339H, S477N). These results indicate a higher variation and high evolutionary pressure related to RBD, impacting the strength of the virus and its interaction with its host and reinforcing the need for a constant monitoring focused on this subdomain [48].

Almost all the positively selected substitutions (21 out of 29) with frequency higher than 90%, were confirmed to be prevalent also in many other different and more recent sub-lineages (JN.1* and its 940 sub-lineages) according to data reported from outbreak.info [14] and shown in Table 3. In addition, according to deep mutational scanning data (summarized in Table 3) 17 high frequency (>90%) mutations provide an immune escape (IE) effect to the Spike protein while only four, located exclusively in the RBD subdomain, appear to influence ACE2 binding affinity. As mentioned above, this data supports the importance of identifying potential “marker sites” which are valid to cover numerous lineages with important implications for vaccine formulations and diagnostics mainly when it is backed up with experimentally proven results like the deep mutational scanning outcomes [13,44–46,49–57]. It is important to mention that protein stability inferred in-silico provided additional input when combined with the other data (Table 3) as it could identify an increased or a decreased influence of each specific replacement when compared to the wild-type. However, the method could be considered as complementary asset to laboratory experiments as it is based on statistical assumptions in the protein three dimensional space and its crystalized conformation [58].

Similar variability was also observed from mutation occurrence and frequency overall trend in all datasets (Table 1), where RBD subdomain represented the highest number of shared mutations between Omicron, XBB*, EG* and BA* sub-lineages. In addition, RBD contained also one conserved mutation (N501Y) along all analysed variants, involved in immune evasion and in the binding affinity to ACE2 [59,60]. On the other hand, in NTD subdomain two mutations (A27S, G142D) became conserved with high frequency (>65%) eventhough their appearance in Delta variant seemed to be sporadic (frequency 0.5%). This highlights the importance of regularly monitor not only conserved mutations but also those less frequent mainly if they are involved in important function for virus survival. An additional important mutation shared by all variants is the D614G. Previous studies reported its multiple effects on Spike protein, including the enhancement of viral infectivity and transmission and its binding affinity to ACE2 by altering the conformation of the RBD subdomain [61,62]. In S2 subunit the prevalent high frequency mutations, appeared in Omicron and remained conserved in EG*, XBB* and BA* sub-lineages, fall either in HR1 subdomain (Q954H and N969K) associated with decreased protein stability [63] and S1in S2 subunit (N764K and D796Y) involved in an enhanced infectivity and transmission of the virus [64]. Many other a.a. substitutions here identified were also previously reported in literature. In particular, the V83A, R346T, together with N460K (the latter identified in our study at frequency of 72%) were reported to have a role in increasing fusogenicity and infectivity [28,65].

Importantly, two a.a. substitutions F486V and F486L in XBB*, previously reported to confer resistance to antiviral therapies in UK data (COG.UK), were found in our study at low frequency (0.05). This highlights the importance of monitoring even rare mutations especially if they are involved in drug resistance function. It is worth mentioning that in XBB* dataset a high number of low frequency mutations were detected (S3 Table) either due to the higher number of included sequences or to the higher variability of this variant as it is considered one of the variants most influencing the immune escape mechanism [4]. Accordingly, as reported in Table 3 many low frequency mutations were confirmed under positive selection pressure by different methods and at the same time they showed an important immune escape activity (22 in XBB*, two in EG*, two in BA*) and an enhanced binding affinity to ACE2 (five in XBB* and one in EG*) [44–46]. In addition, some known substitutions were not detected in Alpha, Delta and Omicron due to the relatively low number of sequences considered in this analysis as they consisted only of the representatives provided by the source database [30,31]. However, the elaboration and analysis of the a.a. changes already discovered in previous and well-studied variants falls out of the scope of this work.

Many deletions and insertions, either alone or in connection with specific mutations, could play an important role in virus survival. In this study, almost all indels were detected in the NTD subdomain (Fig 2 & Table 2). Although only few of these variations are well known [15,18,38,66,67] we assume that their function can be associated to the involvement of NTD in the viral immune escape and its binding affinity to ACE2 [15,16] and consequently an increased infectivity. However, additional investigations are needed to highlight the role of indels in the virus life cycle and their putative interactions with its host [56].

The approach here described constitutes an alternative method to explore not only SARS-CoV-2 variants, but it could be extended to many other disease-causing organisms as it includes key analytical process combining mutational pattern and evolutionary information. Accordingly, data processing pipeline can be tested, optimized and standardized to be executed on expanded computational infrastructures to satisfy the requisites to conduct large-scale studies needed to monitor new genomic data or to cope with newly appearing disease outbreak.

## Supporting information

S1 Table Spike mutations in XBB. 1.5*, XBB.1.9*, XBB.1.16*, XBB.2.3*, FE.1*, EG.5*, JN.1* as reported by outbreak.info. Mutations in at least 75% of the sequences.
(DOCX)

S2 Table Number of sequences for each lineage/sub-lineage for XBB* (a), EG* (b) and BA * (c) datasets. (DOCX)

S1 Fig Maximum likelihood phylogenetic Tree of Spike protein gene obtained from IQTREE. The tree includes 686 leaves corresponding to Alpha, Delta, Omicron, XBB*. EG* and BA* variants and the reference strain Wuhan-Hu-1 (NC_045512.2). Nodes supports were obtained with ultra-fast nonparametric bootstrap analysis (available from IQTREE package) with 1000 replicates.
(PDF)

S3 Table Amino acid substitutions (a.a. ) frequencies of Spike protein coding gene of Alpha, Delta, Omicron, XBB*, EG*, BA* variants with Wuhan-Hu-1 (NC_045512.2) as reference. Each a.a. replacement was attributed to the functional Spike subunits and the corresponding subdomain. Frequencies are reported in percentage scale.
(DOCX)

S2 Fig High frequency (>10%) Spike gene amino acid substitutions sites. The figure shows frequencies higher than 10% in at least one of the analyzed variants. Nearly 85% (77 out of 91) of these high-frequency mutations are found in the S1 subunit, distributed as follows: 29 in the NTD subdomain, 37 in the RBD, and 11 in the region between S1 and S2. Within the S2 subunit, most mutations are concentrated in the HR1 subdomain, with a single mutation observed in the fusion peptide (FP) and another in HR2. Crucially, the N501Y and D614G substitutions are present in all variants, while A27S and G142D are common to five variants, excluding Alpha. S1: Subunit 1 (14–685 residues): NTD: N-terminal subdomain (14–305 residues), RBD: C-terminal receptor binding subdomain (319–541 residues). S2: Subunit 2 (686–1273 residues): FP: Fusion peptide (788–806 residues), HR1: Heptapeptide repeat sequence 1 (912–984 residues), HR2: Heptapeptide repeat sequence 2 (1163–1213 residues).
(DOCX)

S4 Table Amino acid deletions frequencies of Spike protein coding gene of Alpha, Delta, Omicron, XBB*, EG*, BA* variants with Wuhan-Hu-1 (NC_045512.2) as reference. Each deletion was attributed to the functional Spike subunits and the corresponding subdomain. Frequencies are reported in percentage scale.
(DOCX)
