# Supplementary material for: Investigating the evolutionary dynamics and mutational pattern of SARS-CoV-2 spike gene on selected SARS-CoV-2 variants
Source: PLoS One. 2025 Oct 21;20(10):e0333093. doi: 10.1371/journal.pone.0333093 (PMC12539718; doi:10.1371/journal.pone.0333093)
Supplement: S1 Table — 1.5*, XBB.1.9*, XBB.1.16*, XBB.2.3*, FE.1*, EG.5*, JN.1* as reported by outbreak.info. Mutations in at least 75% of the sequences. (DOCX) [file pone.0333093.s001.docx]

**Table S1.** Spike mutations in XBB.1.5*, XBB.1.9*, XBB.1.16*, XBB.2.3*, FE.1*, EG.5*, JN.1* as reported by outbreak.info. Mutations in at least 75% of the sequences.

^#^ some mutations were selected only in specific sub lineages.

| **Spike mutations XBB.1.5*** | **Spike mutations XBB.1.9*** | **Spike mutations XBB.1.16*** | **Spike mutations XBB.2.3*** | **^#^Spike mutations FE.1*** | **Spike mutations EG.5*** | **Spike mutations BA.2.86*** | **Spike mutations**  **JN.1*** |
| --- | --- | --- | --- | --- | --- | --- | --- |
| T19I | T19I | T19I | T19I | T19I | T19I | T19I | T19I |
| L24S | L24S | L24S | L24S | L24S | L24S | R21T | R21T |
| del25/27 | del25/27 | del25/27 | del25/27 | del25/27 | del25/27 | L24S | L24S |
| V83A | V83A | V83A | V83A | V83A | Q52H | del25/27 | del25/27 |
| G142D | G142D | G142D | G142D | G142D | V83A | S50L | S50L |
| del144/144 | del144/144 | del144/144 | del144/144 | del144/144 | G142D | del69/70 | del69/70 |
| H146Q | H146Q | H146Q | H146Q | H146Q | del144/144 | V127F | V127F |
| Q183E | Q183E | E180V | Q183E | Q183E | H146Q | G142D | G142D |
| V213E | V213E | Q183E | V213E | V213E | Q183E | del144/144 | del144/144 |
| G252V | G252V | V213E | D253G | G252V | V213E | F157S | F157S |
| G339H | G339H | G252V | G339H | G339H | G252V | R158G | R158G |
| R346T | R346T | G339H | R346T | R346T | G339H | N211I | N211I |
| L368I | L368I | R346T | L368I | L368I | R346T | del212/212 | del212/212 |
| S371F | S371F | L368I | S371F | S371F | L368I | V213G | V213G |
| S373P | S373P | S371F | S373P | S373P | S371F | L216F | L216F |
| S375F | S375F | S373P | S375F | S375F | S373P | H245N | H245N |
| T376A | T376A | S375F | T376A | T376A | S375F | A264D | A264D |
| D405N | D405N | T376A | D405N | D405N | T376A | I332V | I332V |
| R408S | R408S | D405N | R408S | R408S | D405N | G339H | G339H |
| K417N | K417N | R408S | K417N | K417N | R408S | K356T | K356T |
| N440K | N440K | K417N | N440K | N440K | K417N | S371F | S371F |
| V445P | V445P | N440K | V445P | V445P | N440K | S373P | S373P |
| G446S | G446S | V445P | G446S | G446S | V445P | S375F | S375F |
| N460K | N460K | G446S | N460K | F456L | G446S | T376A | T376A |
| S477N | S477N | N460K | S477N | N460K | F456L | R403K | R403K |
| T478K | T478K | S477N | E484A | S477N | N460K | D405N | D405N |
| E484A | E484A | T478R | F486P | T478K | S477N | R408S | R408S |
| F486P | F486P | E484A | F490S | E484A | T478K | K417N | K417N |
| F490S | F490S | F486P | Q498R | F486P | E484A | N440K | N440K |
| Q498R | Q498R | F490S | N501Y | F490S | F486P | V445H | V445H |
| N501Y | N501Y | Q498R | Y505H | Q498R | F490S | G446S | G446S |
| Y505H | Y505H | N501Y | P521S | N501Y | Q498R | N450D | N450D |
| D614G | D614G | Y505H | D614G | Y505H | N501Y | L452W | L452W |
| H655Y | H655Y | D614G | H655Y | D614G | Y505H | L455S | L455S |
| N679K | N679K | H655Y | N679K | E554K | D614G | N460K | N460K |
| P681H | P681H | N679K | P681H | H655Y | H655Y | S477N | S477N |
| N764K | N764K | P681H | N764K | N679K | N679K | T478K | T478K |
| D796Y | D796Y | N764K | D796Y | P681H | P681H | N481K | N481K |
| Q954H | Q954H | D796Y | Q954H | N764K | N764K | del483/483 | del483/483 |
| N969K | N969K | Q954H | N969K | D796Y | D796Y | E484K | E484K |
|  |  | N969K |  | Q954H | Q954H | F486P | F486P |
|  |  |  |  | N969K | N969K | Q498R | Q498R |
|  |  |  |  | K1086R |  | N501Y | N501Y |
|  |  |  |  |  |  | Y505H | Y505H |
|  |  |  |  |  |  | E554K | E554K |
|  |  |  |  |  |  | A570V | A570V |
|  |  |  |  |  |  | D614G | D614G |
|  |  |  |  |  |  | P621S | P621S |
|  |  |  |  |  |  | H655Y | H655Y |
|  |  |  |  |  |  | N679K | N679K |
|  |  |  |  |  |  | P681R | P681R |
|  |  |  |  |  |  | N764K | N764K |
|  |  |  |  |  |  | D796Y | D796Y |
|  |  |  |  |  |  | S939F | S939F |
|  |  |  |  |  |  | Q954H | Q954H |
|  |  |  |  |  |  | N969K | N969K |
|  |  |  |  |  |  | P1143L | P1143L |
