# Supplementary material for: Investigating the evolutionary dynamics and mutational pattern of SARS-CoV-2 spike gene on selected SARS-CoV-2 variants
Source: PLoS One. 2025 Oct 21;20(10):e0333093. doi: 10.1371/journal.pone.0333093 (PMC12539718; doi:10.1371/journal.pone.0333093)
Supplement: S2 Table — (DOCX) [file pone.0333093.s002.docx]

**Tables S2.** Number of sequences for each lineage/sub-lineage for XBB* (a), EG* (b) and BA * (c) datasets.

**a)**

| **Lineages /sub-lineages** | **Number of sequences** |
| --- | --- |
| XBB.1.5 | 1815 |
| XBB.1.5.1 | 14 |
| XBB.1.5.11 | 32 |
| XBB.1.5.12 | 65 |
| XBB.1.5.13 | 129 |
| XBB.1.5.14 | 30 |
| XBB.1.5.15 | 31 |
| XBB.1.5.16 | 21 |
| XBB.1.5.17 | 17 |
| XBB.1.5.18 | 9 |
| XBB.1.5.19 | 27 |
| XBB.1.5.20 | 22 |
| XBB.1.5.21 | 32 |
| XBB.1.5.23 | 26 |
| XBB.1.5.24 | 49 |
| XBB.1.5.25 | 3 |
| XBB.1.5.26 | 1 |
| XBB.1.5.28 | 6 |
| XBB.1.5.3 | 2 |
| XBB.1.5.31 | 2 |
| XBB.1.5.32 | 8 |
| XBB.1.5.33 | 3 |
| XBB.1.5.35 | 10 |
| XBB.1.5.36 | 9 |
| XBB.1.5.37 | 41 |
| XBB.1.5.38 | 38 |
| XBB.1.5.39 | 42 |
| XBB.1.5.4 | 10 |
| XBB.1.5.40 | 14 |
| XBB.1.5.43 | 1 |
| XBB.1.5.46 | 45 |
| XBB.1.5.47 | 7 |
| XBB.1.5.48 | 14 |
| XBB.1.5.49 | 14 |
| XBB.1.5.5 | 3 |
| XBB.1.5.51 | 2 |
| XBB.1.5.52 | 17 |
| XBB.1.5.55 | 2 |
| XBB.1.5.56 | 1 |
| XBB.1.5.57 | 5 |
| XBB.1.5.59 | 14 |
| XBB.1.5.6 | 6 |
| XBB.1.5.61 | 3 |
| XBB.1.5.62 | 6 |
| XBB.1.5.63 | 13 |
| XBB.1.5.65 | 27 |
| XBB.1.5.66 | 8 |
| XBB.1.5.67 | 13 |
| XBB.1.5.68 | 2 |
| XBB.1.5.69 | 1 |
| XBB.1.5.7 | 97 |
| XBB.1.5.9 | 1 |
| XBB.1.9.1 | 497 |
| XBB.1.9.2 | 173 |
| XBB.1.16 | 95 |
| XBB.1.16.1 | 10 |
| XBB.1.16.2 | 5 |
| XBB.1.16.3 | 2 |
| XBB.2.3 | 59 |
| XBB.2.3.1 | 1 |
| XBB.2.3.11 | 8 |
| XBB.2.3.2 | 8 |
| XBB.2.3.3 | 18 |
| XBB.2.3.4 | 1 |
| XBB.2.3.5 | 1 |
| XBB.2.3.6 | 1 |
| FE.1 | 8 |
| FE.1.1 | 18 |
| FE.1.2 | 9 |
| **Total** | **3724** |

**b)**

| **Lineages/sub-lineages** | **Number of sequences** |
| --- | --- |
| EG.5 | 15 |
| EG.5.1 | 120 |
| EG.5.1.1 | 160 |
| EG.5.1.2 | 1 |
| EG.5.1.3 | 74 |
| EG.5.1.4 | 32 |
| EG.5.1.5 | 4 |
| EG.5.1.6 | 24 |
| EG.5.2 | 3 |
| EG.5.2.3 | 3 |
| **Total** | **436** |

**c)**

| **Lineages/sub-lineages** | **Number of sequences** |
| --- | --- |
| BA.2.86 | 32 |
| BA.2.86.1 | 176 |
| **Total** | **208** |
