# Supplementary material for: Investigating the evolutionary dynamics and mutational pattern of SARS-CoV-2 spike gene on selected SARS-CoV-2 variants
Source: PLoS One. 2025 Oct 21;20(10):e0333093. doi: 10.1371/journal.pone.0333093 (PMC12539718; doi:10.1371/journal.pone.0333093)
Supplement: S1 Fig — The tree includes 686 leaves corresponding to Alpha, Delta, Omicron, XBB*. EG* and BA* variants and the reference strain Wuhan-Hu-1 (NC_045512.2). Nodes supports were obtained with ultra-fast nonparametric bootstrap analysis (available from IQTREE package) with 1000 replicates. (PDF) [file pone.0333093.s003.pdf]

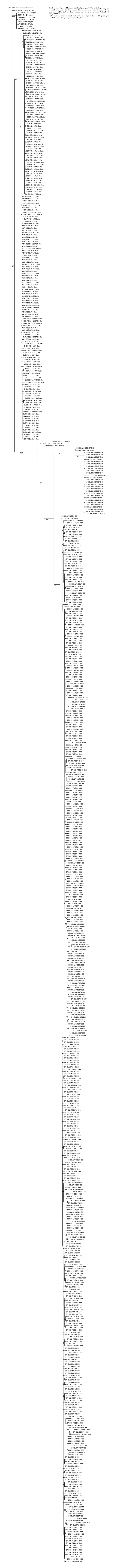

Supplementary Figure 1. Maximum likelihood phylogenetic Tree of Spike protein gene obtained from Iqtree. The tree includes 686 leaves corresponding to Alpha, Delta, Omicrom, XBB*. EG* and BA* variants and the reference strain Wuhan-Hu-1 (NC_045512.2).
Nodes supports were obtained with ultra-fast nonparametric bootstrap analysis (available from Iqtree package) with 1000 replicates.
