# Supplementary material for: Investigating the evolutionary dynamics and mutational pattern of SARS-CoV-2 spike gene on selected SARS-CoV-2 variants
Source: PLoS One. 2025 Oct 21;20(10):e0333093. doi: 10.1371/journal.pone.0333093 (PMC12539718; doi:10.1371/journal.pone.0333093)
Supplement: S3 Table — ) frequencies of Spike protein coding gene of Alpha, Delta, Omicron, XBB*, EG*, BA* variants with Wuhan-Hu-1 (NC_045512.2) as reference. Each a.a. replacement was attributed to the functional Spike subunits and the corresponding subdomain. Frequencies are reported in percentage scale. (DOCX) [file pone.0333093.s004.docx]

**Table S3.** Amino acid substitutions (a.a.) frequencies of spike protein coding gene of Alpha, Delta, Omicron, XBB*, EG*, BA* variants with Wuhan-Hu-1 (NC_045512.2) as reference.
Each a.a. replacement was attributed to the functional Spike subunits and the corresponding subdomain.
Frequencies are reported in percentage scale.

| **Site** | **Substitution** | **EG*-Frequency** | **BA*-Frequency** | **XBB*-Frequency** | **Delta-Frequency** | **Alpha-Frequency** | **Omicron-Frequency** | **Subunits** | **Subdomains** |
| --- | --- | --- | --- | --- | --- | --- | --- | --- | --- |
| 5 | L-F | 0.688073394495 | NA | 1.02040816327 | 1.10497237569 | NA | NA | SP |  |
| 6 | V-L | NA | NA | 0.0268528464017 | NA | NA | NA | SP |  |
| 9 | P-L | NA | NA | 0.0537056928034 | NA | NA | NA | SP |  |
| 12 | S-F | NA | NA | 0.0268528464017 | NA | NA | NA | SP |  |
| 14 | Q-H | 0.229357798165 | NA | 0.0537056928034 | NA | NA | NA | S1 |  |
| 14 | Q-K | 0.229357798165 | NA | NA | NA | NA | NA | S1 |  |
| 14 | Q-R | NA | NA | 0.0268528464017 | NA | NA | NA | S1 |  |
| 15 | C-R | NA | NA | 0.0268528464017 | NA | NA | NA | S1 |  |
| 16 | V-F | NA | NA | 0.0268528464017 | NA | NA | NA | S1 |  |
| 16 | V-I | 0.45871559633 | NA | 0.0268528464017 | NA | NA | NA | S1 |  |
| 17 | N-D | NA | NA | 0.0268528464017 | NA | NA | NA | S1 |  |
| 19 | T-I | 95.871559633 | 79.3269230769 | 99.597207304 | NA | NA | 100 | S1 | NTD |
| 19 | T-L | 2.29357798165 | NA | 0.0537056928034 | NA | NA | NA | S1 | NTD |
| 19 | T-R | NA | NA | NA | 100 | NA | NA | S1 | NTD |
| 19 | T-V | NA | NA | 0.0537056928034 | NA | NA | NA | S1 | NTD |
| 21 | R-T | NA | 76.9230769231 | NA | 1.10497237569 | NA | NA | S1 | NTD |
| 22 | T-I | NA | NA | 0.0268528464017 | 0.552486187845 | NA | NA | S1 | NTD |
| 24 | L-S | NA | NA | 0.214822771214 | NA | NA | NA | S1 | NTD |
| 25 | P-Y | NA | NA | 0.214822771214 | NA | NA | NA | S1 | NTD |
| 26 | P-S | NA | NA | NA | 1.10497237569 | NA | NA | S1 | NTD |
| 26 | P-T | NA | NA | 0.214822771214 | NA | NA | NA | S1 | NTD |
| 27 | A-S | 98.3944954128 | 86.5384615385 | 99.4092373792 | 0.552486187845 | NA | 66.6666666667 | S1 | NTD |
| 28 | Y-H | NA | NA | 0.0268528464017 | NA | NA | NA | S1 | NTD |
| 29 | T-I | NA | NA | 0.0805585392052 | NA | NA | NA | S1 | NTD |
| 29 | T-S | NA | NA | 0.0537056928034 | NA | NA | NA | S1 | NTD |
| 31 | S-F | NA | NA | 0.0537056928034 | NA | NA | NA | S1 | NTD |
| 32 | F-L | NA | NA | 0.0268528464017 | NA | NA | NA | S1 | NTD |
| 32 | F-S | NA | NA | 0.0537056928034 | NA | NA | NA | S1 | NTD |
| 36 | V-F | NA | NA | NA | 0.552486187845 | NA | NA | S1 | NTD |
| 37 | Y-S | 0.229357798165 | NA | NA | NA | NA | NA | S1 | NTD |
| 38 | Y-H | NA | NA | 0.0268528464017 | NA | NA | NA | S1 | NTD |
| 49 | H-Y | NA | NA | 0.0537056928034 | NA | NA | NA | S1 | NTD |
| 50 | S-L | NA | 98.5576923077 | NA | NA | NA | NA | S1 | NTD |
| 51 | T-I | NA | NA | 0.107411385607 | NA | NA | NA | S1 | NTD |
| 52 | Q-H | 78.4403669725 | NA | 0.0268528464017 | NA | NA | NA | S1 | NTD |
| 53 | D-N | NA | NA | 0.0268528464017 | NA | NA | NA | S1 | NTD |
| 54 | L-F | NA | NA | 0.0268528464017 | NA | NA | NA | S1 | NTD |
| 55 | F-Y | NA | NA | 0.0268528464017 | NA | NA | NA | S1 | NTD |
| 59 | F-L | NA | NA | 0.0537056928034 | NA | NA | NA | S1 | NTD |
| 62 | V-I | NA | NA | 0.0268528464017 | NA | NA | NA | S1 | NTD |
| 64 | W-R | NA | NA | 0.0537056928034 | NA | NA | NA | S1 | NTD |
| 66 | H-Q | NA | NA | 0.0268528464017 | NA | NA | NA | S1 | NTD |
| 67 | A-S | NA | NA | NA | 0.552486187845 | NA | NA | S1 | NTD |
| 67 | A-V | 0.45871559633 | NA | 0.107411385607 | NA | NA | 33.3333333333 | S1 | NTD |
| 69 | H-R | NA | NA | 0.0268528464017 | NA | NA | NA | S1 | NTD |
| 69 | H-Y | NA | NA | 0.0268528464017 | NA | NA | NA | S1 | NTD |
| 70 | V-A | NA | NA | 0.0268528464017 | NA | NA | NA | S1 | NTD |
| 70 | V-I | NA | 2.88461538462 | NA | NA | NA | NA | S1 | NTD |
| 71 | S-A | NA | NA | 0.0805585392052 | NA | NA | NA | S1 | NTD |
| 71 | S-F | NA | NA | 0.0537056928034 | NA | NA | NA | S1 | NTD |
| 72 | G-E | NA | NA | 0.0268528464017 | NA | NA | NA | S1 | NTD |
| 73 | T-I | NA | NA | NA | NA | 12.5 | NA | S1 | NTD |
| 74 | N-S | 0.229357798165 | NA | NA | NA | NA | NA | S1 | NTD |
| 74 | N-Y | NA | NA | 0.0268528464017 | NA | NA | NA | S1 | NTD |
| 75 | G-V | 1.14678899083 | NA | 0.107411385607 | NA | NA | NA | S1 | NTD |
| 76 | T-A | 0.229357798165 | NA | NA | NA | NA | NA | S1 | NTD |
| 76 | T-I | NA | NA | 0.0268528464017 | NA | NA | NA | S1 | NTD |
| 76 | T-N | NA | NA | 0.107411385607 | NA | NA | NA | S1 | NTD |
| 77 | K-N | 0.229357798165 | NA | 0.0537056928034 | NA | NA | NA | S1 | NTD |
| 77 | K-T | NA | NA | NA | 1.10497237569 | NA | NA | S1 | NTD |
| 78 | R-K | NA | NA | 0.0537056928034 | NA | NA | NA | S1 | NTD |
| 78 | R-S | NA | NA | NA | 0.552486187845 | NA | NA | S1 | NTD |
| 80 | D-E | NA | NA | 0.0268528464017 | NA | NA | NA | S1 | NTD |
| 80 | D-N | NA | NA | 0.0268528464017 | NA | NA | NA | S1 | NTD |
| 80 | D-Y | NA | NA | 0.0805585392052 | NA | NA | NA | S1 | NTD |
| 82 | P-S | NA | NA | 0.0268528464017 | NA | NA | NA | S1 | NTD |
| 83 | V-A | 96.7889908257 | NA | 94.7368421053 | NA | NA | NA | S1 | NTD |
| 85 | P-Y | NA | NA | 0.0268528464017 | NA | NA | NA | S1 | NTD |
| 87 | N-A | NA | NA | 0.0268528464017 | NA | NA | NA | S1 | NTD |
| 88 | D-S | NA | NA | 0.0268528464017 | NA | NA | NA | S1 | NTD |
| 89 | G-T | NA | NA | 0.0268528464017 | NA | NA | NA | S1 | NTD |
| 90 | V-I | NA | NA | 0.0268528464017 | NA | NA | NA | S1 | NTD |
| 91 | Y-V | NA | NA | 0.0268528464017 | NA | NA | NA | S1 | NTD |
| 92 | F-C | NA | NA | 0.0268528464017 | NA | NA | NA | S1 | NTD |
| 93 | A-S | NA | NA | 0.214822771214 | NA | NA | NA | S1 | NTD |
| 94 | S-F | NA | NA | 0.0268528464017 | NA | NA | NA | S1 | NTD |
| 95 | T-I | NA | 1.92307692308 | 0.0268528464017 | 39.226519337 | NA | 33.3333333333 | S1 | NTD |
| 96 | E-A | NA | NA | 0.0537056928034 | NA | NA | NA | S1 | NTD |
| 97 | K-E | NA | NA | 0.0268528464017 | NA | NA | NA | S1 | NTD |
| 97 | K-N | 0.45871559633 | NA | NA | NA | NA | NA | S1 | NTD |
| 97 | K-T | NA | NA | 0.0805585392052 | NA | NA | NA | S1 | NTD |
| 98 | S-F | 0.229357798165 | NA | 0.214822771214 | NA | NA | NA | S1 | NTD |
| 105 | I-L | NA | NA | 0.0268528464017 | NA | NA | NA | S1 | NTD |
| 111 | D-E | NA | NA | 0.0268528464017 | NA | NA | NA | S1 | NTD |
| 112 | S-L | NA | NA | NA | 0.552486187845 | NA | NA | S1 | NTD |
| 113 | K-N | NA | NA | 0.0268528464017 | NA | NA | NA | S1 | NTD |
| 120 | V-L | NA | NA | 0.0268528464017 | NA | NA | NA | S1 | NTD |
| 127 | V-F | NA | 99.5192307692 | NA | NA | NA | NA | S1 | NTD |
| 128 | I-V | NA | NA | 0.187969924812 | NA | NA | NA | S1 | NTD |
| 137 | N-M | NA | NA | 0.0268528464017 | NA | NA | NA | S1 | NTD |
| 138 | D-Q | NA | NA | 0.0537056928034 | NA | NA | NA | S1 | NTD |
| 139 | P-L | NA | NA | 0.0268528464017 | NA | NA | NA | S1 | NTD |
| 139 | P-V | NA | NA | 0.0268528464017 | NA | NA | NA | S1 | NTD |
| 140 | F-L | NA | NA | 0.0537056928034 | NA | NA | NA | S1 | NTD |
| 141 | L-F | NA | NA | 0.0537056928034 | NA | NA | NA | S1 | NTD |
| 142 | G-D | 97.4770642202 | 97.5961538462 | 98.4425349087 | 100 | NA | 100 | S1 | NTD |
| 144 | Y-L | NA | NA | 0.0268528464017 | NA | NA | NA | S1 | NTD |
| 145 | Y-D | 0.229357798165 | NA | 0.0537056928034 | NA | NA | NA | S1 | NTD |
| 145 | Y-H | NA | NA | 0.0268528464017 | 1.65745856354 | NA | NA | S1 | NTD |
| 145 | Y-Q | NA | NA | 0.0537056928034 | NA | NA | NA | S1 | NTD |
| 146 | H-K | 4.12844036697 | NA | 5.20945220193 | NA | NA | NA | S1 | NTD |
| 146 | H-P | 0.229357798165 | NA | 0.107411385607 | NA | NA | NA | S1 | NTD |
| 146 | H-Q | 94.7247706422 | NA | 93.9312567132 | NA | NA | NA | S1 | NTD |
| 147 | K-N | NA | NA | 0.0537056928034 | NA | NA | NA | S1 | NTD |
| 148 | N-K | NA | NA | 0.0268528464017 | NA | NA | NA | S1 | NTD |
| 148 | N-T | 0.45871559633 | NA | 0.16111707841 | NA | NA | NA | S1 | NTD |
| 150 | K-N | NA | 0.480769230769 | 0.0537056928034 | NA | NA | NA | S1 | NTD |
| 151 | S-I | NA | NA | 0.134264232009 | NA | NA | NA | S1 | NTD |
| 152 | W-L | NA | NA | 0.107411385607 | NA | NA | NA | S1 | NTD |
| 152 | W-R | NA | NA | 0.0537056928034 | NA | NA | NA | S1 | NTD |
| 153 | M-I | NA | NA | 0.0268528464017 | NA | NA | NA | S1 | NTD |
| 153 | M-T | 0.45871559633 | NA | 0.0268528464017 | NA | NA | NA | S1 | NTD |
| 154 | E-A | 0.229357798165 | NA | NA | NA | NA | NA | S1 | NTD |
| 156 | E-G | NA | NA | NA | 98.3425414365 | NA | NA | S1 | NTD |
| 156 | E-K | NA | NA | 0.0268528464017 | NA | NA | NA | S1 | NTD |
| 157 | F-L | 4.81651376147 | NA | 0.107411385607 | NA | NA | NA | S1 | NTD |
| 157 | F-S | NA | 97.5961538462 | NA | NA | NA | NA | S1 | NTD |
| 158 | R-G | NA | 97.5961538462 | NA | NA | NA | NA | S1 | NTD |
| 158 | R-T | NA | NA | 0.0537056928034 | NA | NA | NA | S1 | NTD |
| 164 | N-T | NA | NA | 0.0268528464017 | NA | NA | NA | S1 | NTD |
| 173 | Q-H | NA | NA | NA | 1.10497237569 | NA | NA | S1 | NTD |
| 173 | Q-K | NA | NA | 0.0268528464017 | NA | NA | NA | S1 | NTD |
| 173 | Q-R | NA | NA | 0.0268528464017 | NA | NA | NA | S1 | NTD |
| 174 | P-H | NA | NA | 0.0268528464017 | NA | NA | NA | S1 | NTD |
| 174 | P-S | NA | NA | NA | 0.552486187845 | NA | NA | S1 | NTD |
| 175 | F-L | 0.45871559633 | NA | NA | NA | NA | NA | S1 | NTD |
| 176 | L-F | 0.688073394495 | NA | 0.0537056928034 | NA | NA | NA | S1 | NTD |
| 176 | L-P | 0.45871559633 | NA | NA | NA | NA | NA | S1 | NTD |
| 177 | M-I | NA | NA | 0.0805585392052 | 0.552486187845 | NA | NA | S1 | NTD |
| 177 | M-T | NA | NA | 0.0268528464017 | NA | NA | NA | S1 | NTD |
| 178 | D-H | NA | NA | 0.0268528464017 | NA | NA | NA | S1 | NTD |
| 178 | D-N | 0.229357798165 | NA | 0.0268528464017 | NA | NA | NA | S1 | NTD |
| 180 | E-K | NA | NA | 0.0268528464017 | NA | NA | NA | S1 | NTD |
| 180 | E-Q | NA | NA | 0.0537056928034 | NA | NA | NA | S1 | NTD |
| 180 | E-V | NA | NA | 2.98066595059 | NA | NA | NA | S1 | NTD |
| 181 | G-R | NA | NA | 0.0268528464017 | NA | NA | NA | S1 | NTD |
| 181 | G-V | 0.45871559633 | NA | 0.0268528464017 | NA | NA | NA | S1 | NTD |
| 182 | K-I | NA | NA | 0.0268528464017 | NA | NA | NA | S1 | NTD |
| 182 | K-N | NA | NA | 0.483351235231 | 0.552486187845 | NA | NA | S1 | NTD |
| 182 | K-Q | NA | NA | 0.295381310419 | NA | NA | NA | S1 | NTD |
| 182 | K-R | NA | NA | 0.0268528464017 | NA | NA | NA | S1 | NTD |
| 183 | Q-D | NA | NA | 0.0268528464017 | NA | NA | NA | S1 | NTD |
| 183 | Q-E | 96.7889908257 | NA | 98.4156820623 | NA | NA | NA | S1 | NTD |
| 183 | Q-Z | 0.688073394495 | NA | 0.510204081633 | NA | NA | NA | S1 | NTD |
| 184 | G-S | 2.52293577982 | NA | NA | NA | NA | NA | S1 | NTD |
| 184 | G-V | NA | NA | 0.295381310419 | NA | NA | NA | S1 | NTD |
| 185 | N-D | NA | NA | 0.0268528464017 | NA | NA | NA | S1 | NTD |
| 185 | N-I | NA | NA | 0.0268528464017 | NA | NA | NA | S1 | NTD |
| 185 | N-S | NA | NA | 0.0268528464017 | NA | NA | NA | S1 | NTD |
| 185 | N-T | NA | NA | 0.0268528464017 | NA | NA | NA | S1 | NTD |
| 186 | F-I | 0.229357798165 | NA | NA | NA | NA | NA | S1 | NTD |
| 186 | F-L | NA | NA | 0.0537056928034 | NA | NA | NA | S1 | NTD |
| 187 | K-N | NA | NA | 0.0268528464017 | NA | NA | NA | S1 | NTD |
| 188 | N-D | NA | NA | 0.0268528464017 | NA | NA | NA | S1 | NTD |
| 188 | N-T | NA | NA | 0.0268528464017 | NA | NA | NA | S1 | NTD |
| 189 | L-I | NA | NA | 0.0537056928034 | NA | NA | NA | S1 | NTD |
| 190 | R-K | NA | NA | 0.0268528464017 | NA | NA | NA | S1 | NTD |
| 191 | E-A | 0.229357798165 | NA | NA | NA | NA | NA | S1 | NTD |
| 193 | V-L | NA | 0.480769230769 | 0.0268528464017 | NA | NA | NA | S1 | NTD |
| 197 | I-T | NA | NA | 0.0268528464017 | NA | NA | NA | S1 | NTD |
| 197 | I-V | NA | NA | 0.0537056928034 | NA | NA | NA | S1 | NTD |
| 200 | Y-C | NA | NA | 0.107411385607 | NA | NA | NA | S1 | NTD |
| 208 | T-A | NA | NA | 0.0537056928034 | NA | NA | NA | S1 | NTD |
| 209 | P-L | NA | NA | 0.0268528464017 | NA | NA | NA | S1 | NTD |
| 209 | P-S | NA | NA | 0.0537056928034 | NA | NA | NA | S1 | NTD |
| 211 | N-I | NA | NA | NA | NA | NA | 33.3333333333 | S1 | NTD |
| 211 | N-K | NA | NA | 0.0268528464017 | NA | NA | NA | S1 | NTD |
| 212 | L-I | NA | 99.0384615385 | NA | NA | NA | NA | S1 | NTD |
| 212 | L-V | NA | NA | NA | NA | NA | 33.3333333333 | S1 | NTD |
| 213 | V-E | 99.5412844037 | NA | 99.0870032223 | NA | NA | NA | S1 | NTD |
| 213 | V-G | NA | 100 | 0.16111707841 | NA | NA | 66.6666666667 | S1 | NTD |
| 213 | V-L | NA | NA | NA | 0.552486187845 | NA | NA | S1 | NTD |
| 213 | V-R | NA | NA | NA | NA | NA | 33.3333333333 | S1 | NTD |
| 214 | R-E | NA | NA | NA | NA | NA | 33.3333333333 | S1 | NTD |
| 214 | R-H | NA | NA | 0.0268528464017 | NA | NA | NA | S1 | NTD |
| 214 | R-L | NA | NA | 0.0537056928034 | NA | NA | NA | S1 | NTD |
| 214 | R-P | NA | NA | 0.0268528464017 | NA | NA | NA | S1 | NTD |
| 215 | D-G | NA | NA | 0.0805585392052 | NA | NA | NA | S1 | NTD |
| 215 | D-H | 0.229357798165 | NA | 0.16111707841 | NA | NA | NA | S1 | NTD |
| 215 | D-Y | NA | NA | 0.0537056928034 | NA | NA | NA | S1 | NTD |
| 216 | L-F | NA | 100 | 0.0537056928034 | NA | NA | NA | S1 | NTD |
| 218 | Q-H | NA | NA | NA | 0.552486187845 | NA | NA | S1 | NTD |
| 219 | G-D | 0.229357798165 | NA | NA | NA | NA | NA | S1 | NTD |
| 219 | G-S | NA | NA | NA | 0.552486187845 | NA | NA | S1 | NTD |
| 221 | S-L | NA | NA | 0.241675617615 | NA | NA | NA | S1 | NTD |
| 222 | A-S | 0.229357798165 | NA | 0.107411385607 | NA | NA | NA | S1 | NTD |
| 222 | A-T | NA | NA | 0.0268528464017 | NA | NA | NA | S1 | NTD |
| 222 | A-V | NA | 0.961538461538 | 0.0537056928034 | 18.7845303867 | NA | NA | S1 | NTD |
| 226 | L-F | 0.229357798165 | NA | NA | NA | NA | NA | S1 | NTD |
| 229 | L-F | NA | NA | 0.0268528464017 | NA | NA | NA | S1 | NTD |
| 230 | P-S | NA | NA | 0.0268528464017 | NA | NA | NA | S1 | NTD |
| 231 | I-M | NA | NA | 0.0268528464017 | NA | NA | NA | S1 | NTD |
| 232 | G-V | NA | NA | 0.0268528464017 | NA | NA | NA | S1 | NTD |
| 237 | R-W | NA | NA | 0.0268528464017 | NA | NA | NA | S1 | NTD |
| 240 | T-I | NA | NA | 0.268528464017 | NA | NA | NA | S1 | NTD |
| 242 | L-F | NA | NA | 0.0268528464017 | NA | NA | NA | S1 | NTD |
| 242 | L-I | NA | NA | 0.0537056928034 | NA | NA | NA | S1 | NTD |
| 244 | L-F | NA | NA | 0.0268528464017 | NA | NA | NA | S1 | NTD |
| 244 | L-S | NA | NA | NA | 0.552486187845 | NA | NA | S1 | NTD |
| 245 | H-N | NA | 99.5192307692 | NA | NA | NA | NA | S1 | NTD |
| 245 | H-Y | NA | NA | 0.134264232009 | 0.552486187845 | NA | NA | S1 | NTD |
| 246 | R-T | NA | NA | 0.0268528464017 | NA | NA | NA | S1 | NTD |
| 247 | S-N | NA | NA | 0.0537056928034 | NA | NA | NA | S1 | NTD |
| 249 | L-F | NA | NA | 0.0268528464017 | NA | NA | NA | S1 | NTD |
| 250 | T-N | 0.229357798165 | NA | 0.0268528464017 | NA | NA | NA | S1 | NTD |
| 250 | T-P | NA | NA | 0.0268528464017 | NA | NA | NA | S1 | NTD |
| 250 | T-S | NA | NA | NA | 0.552486187845 | NA | NA | S1 | NTD |
| 251 | P-L | NA | NA | 0.0268528464017 | 1.10497237569 | NA | NA | S1 | NTD |
| 252 | G-A | NA | NA | 0.0268528464017 | NA | NA | NA | S1 | NTD |
| 252 | G-S | NA | NA | 0.0537056928034 | NA | NA | NA | S1 | NTD |
| 252 | G-V | 88.3027522936 | NA | 90.1181525242 | NA | NA | NA | S1 | NTD |
| 253 | D-A | NA | NA | NA | 0.552486187845 | NA | NA | S1 | NTD |
| 253 | D-G | 2.75229357798 | NA | 2.44360902256 | NA | NA | NA | S1 | NTD |
| 253 | D-Y | NA | NA | 0.0268528464017 | NA | NA | NA | S1 | NTD |
| 254 | S-F | NA | NA | 0.0268528464017 | NA | NA | NA | S1 | NTD |
| 255 | S-F | NA | NA | 0.0537056928034 | 1.10497237569 | NA | NA | S1 | NTD |
| 257 | G-D | NA | NA | 0.0268528464017 | NA | NA | NA | S1 | NTD |
| 257 | G-R | 0.229357798165 | NA | NA | NA | NA | NA | S1 | NTD |
| 257 | G-S | NA | NA | 0.16111707841 | NA | NA | NA | S1 | NTD |
| 257 | G-V | 0.229357798165 | 0.480769230769 | 0.0268528464017 | NA | NA | NA | S1 | NTD |
| 258 | W-C | NA | NA | 0.0268528464017 | NA | NA | NA | S1 | NTD |
| 258 | W-L | NA | NA | 0.0537056928034 | 0.552486187845 | NA | NA | S1 | NTD |
| 258 | W-R | 0.229357798165 | NA | NA | NA | NA | NA | S1 | NTD |
| 261 | G-V | 1.60550458716 | NA | 0.0537056928034 | NA | NA | NA | S1 | NTD |
| 262 | A-S | NA | NA | 0.0805585392052 | NA | NA | NA | S1 | NTD |
| 263 | A-T | NA | 0.961538461538 | NA | NA | NA | NA | S1 | NTD |
| 264 | A-D | NA | 96.1538461538 | NA | NA | NA | NA | S1 | NTD |
| 264 | A-V | NA | NA | 0.0268528464017 | NA | NA | NA | S1 | NTD |
| 266 | Y-C | NA | NA | 0.0268528464017 | NA | NA | NA | S1 | NTD |
| 272 | P-S | NA | NA | 0.0268528464017 | NA | NA | NA | S1 | NTD |
| 274 | T-I | NA | NA | 0.0268528464017 | NA | NA | NA | S1 | NTD |
| 278 | K-E | NA | NA | 0.107411385607 | NA | NA | NA | S1 | NTD |
| 278 | K-N | 0.229357798165 | NA | NA | NA | NA | NA | S1 | NTD |
| 286 | T-I | NA | NA | 0.0537056928034 | NA | NA | NA | S1 | NTD |
| 287 | D-E | NA | NA | 0.0268528464017 | NA | NA | NA | S1 | NTD |
| 287 | D-N | NA | NA | 0.0268528464017 | NA | NA | NA | S1 | NTD |
| 288 | A-V | NA | NA | 0.0268528464017 | NA | NA | NA | S1 | NTD |
| 289 | V-I | NA | NA | NA | 1.10497237569 | NA | NA | S1 | NTD |
| 289 | V-L | NA | NA | 0.0268528464017 | NA | NA | NA | S1 | NTD |
| 293 | L-F | NA | NA | 0.0268528464017 | NA | NA | NA | S1 | NTD |
| 299 | T-I | NA | 3.36538461538 | 0.107411385607 | NA | NA | NA | S1 | NTD |
| 304 | K-E | NA | NA | 0.0268528464017 | NA | NA | NA | S1 | NTD |
| 304 | K-N | 0.229357798165 | NA | NA | NA | NA | NA | S1 | NTD |
| 307 | T-I | NA | NA | 0.187969924812 | NA | NA | NA | S1 |  |
| 308 | V-I | NA | NA | 0.0268528464017 | NA | NA | NA | S1 |  |
| 308 | V-L | NA | NA | NA | 1.10497237569 | NA | NA | S1 |  |
| 322 | P-S | NA | NA | 0.0268528464017 | NA | NA | NA | S1 | RBD |
| 323 | T-I | NA | NA | 1.12781954887 | NA | NA | NA | S1 | RBD |
| 324 | E-K | NA | NA | 0.0268528464017 | NA | NA | NA | S1 | RBD |
| 324 | E-V | NA | NA | 0.0268528464017 | NA | NA | NA | S1 | RBD |
| 329 | F-L | NA | NA | 0.0268528464017 | NA | NA | NA | S1 | RBD |
| 330 | P-S | NA | NA | 0.268528464017 | NA | NA | NA | S1 | RBD |
| 332 | I-V | NA | 97.5961538462 | NA | NA | NA | NA | S1 | RBD |
| 335 | L-F | 0.688073394495 | NA | NA | NA | NA | NA | S1 | RBD |
| 337 | P-H | NA | NA | 0.0268528464017 | NA | NA | NA | S1 | RBD |
| 337 | P-L | NA | NA | 0.0268528464017 | NA | NA | NA | S1 | RBD |
| 337 | P-S | NA | NA | 0.0268528464017 | NA | NA | NA | S1 | RBD |
| 339 | G-D | NA | NA | 0.241675617615 | NA | NA | 100 | S1 | RBD |
| 339 | G-H | 97.9357798165 | 97.1153846154 | 98.3082706767 | NA | NA | NA | S1 | RBD |
| 339 | G-N | NA | NA | 0.0268528464017 | NA | NA | NA | S1 | RBD |
| 339 | G-Y | NA | 0.480769230769 | 0.0268528464017 | NA | NA | NA | S1 | RBD |
| 340 | E-K | NA | NA | 0.0268528464017 | NA | NA | NA | S1 | RBD |
| 340 | E-Q | NA | NA | 0.268528464017 | NA | NA | NA | S1 | RBD |
| 341 | V-I | NA | NA | 0.0268528464017 | NA | NA | NA | S1 | RBD |
| 346 | R-I | NA | NA | 0.0537056928034 | NA | NA | NA | S1 | RBD |
| 346 | R-S | NA | NA | 0.0805585392052 | NA | NA | NA | S1 | RBD |
| 346 | R-T | 97.4770642202 | NA | 98.3888292159 | NA | NA | NA | S1 | RBD |
| 348 | A-S | NA | NA | 0.0537056928034 | NA | NA | NA | S1 | RBD |
| 348 | A-T | NA | NA | 0.0268528464017 | NA | NA | NA | S1 | RBD |
| 352 | A-S | NA | NA | 0.0537056928034 | NA | NA | NA | S1 | RBD |
| 352 | A-V | NA | NA | 0.107411385607 | NA | NA | NA | S1 | RBD |
| 354 | N-K | NA | NA | 0.0268528464017 | NA | NA | NA | S1 | RBD |
| 356 | K-E | NA | NA | 0.0268528464017 | NA | NA | NA | S1 | RBD |
| 356 | K-N | NA | NA | 0.0805585392052 | NA | NA | NA | S1 | RBD |
| 356 | K-T | NA | 93.75 | 0.0268528464017 | NA | NA | NA | S1 | RBD |
| 360 | N-K | NA | NA | 0.0268528464017 | NA | NA | NA | S1 | RBD |
| 367 | V-F | NA | NA | 0.0537056928034 | NA | NA | NA | S1 | RBD |
| 367 | V-L | NA | NA | NA | 0.552486187845 | NA | NA | S1 | RBD |
| 368 | L-I | 91.7431192661 | NA | 94.6831364125 | NA | NA | NA | S1 | RBD |
| 368 | L-J | 0.229357798165 | NA | 0.0268528464017 | NA | NA | NA | S1 | RBD |
| 371 | S-F | 92.6605504587 | 94.2307692308 | 95.9183673469 | NA | NA | 33.3333333333 | S1 | RBD |
| 371 | S-L | NA | NA | NA | NA | NA | 66.6666666667 | S1 | RBD |
| 372 | A-V | NA | NA | 0.0268528464017 | NA | NA | NA | S1 | RBD |
| 373 | S-P | 95.1834862385 | 95.6730769231 | 96.2137486574 | NA | NA | 100 | S1 | RBD |
| 373 | S-Q | NA | NA | 0.0537056928034 | NA | NA | NA | S1 | RBD |
| 374 | F-L | NA | NA | 0.0537056928034 | NA | NA | NA | S1 | RBD |
| 375 | S-A | 0.229357798165 | NA | NA | NA | NA | NA | S1 | RBD |
| 375 | S-F | 95.1834862385 | 95.1923076923 | 95.6498388829 | NA | NA | 100 | S1 | RBD |
| 376 | T-A | 94.9541284404 | 95.1923076923 | 93.9044038668 | NA | NA | 33.3333333333 | S1 | RBD |
| 376 | T-P | 0.229357798165 | NA | NA | NA | NA | NA | S1 | RBD |
| 382 | V-L | NA | NA | 0.16111707841 | NA | NA | NA | S1 | RBD |
| 385 | T-I | NA | NA | 0.107411385607 | NA | NA | NA | S1 | RBD |
| 395 | V-E | NA | NA | 0.0268528464017 | NA | NA | NA | S1 | RBD |
| 397 | A-V | NA | 0.480769230769 | NA | NA | NA | NA | S1 | RBD |
| 399 | S-T | NA | NA | 0.0268528464017 | NA | NA | NA | S1 | RBD |
| 403 | R-K | NA | 69.7115384615 | 0.0268528464017 | NA | NA | NA | S1 | RBD |
| 405 | D-B | NA | NA | NA | NA | NA | 33.3333333333 | S1 | RBD |
| 405 | D-N | 94.2660550459 | 95.6730769231 | 95.8109559613 | NA | NA | 33.3333333333 | S1 | RBD |
| 407 | V-G | NA | NA | 0.107411385607 | NA | NA | NA | S1 | RBD |
| 408 | R-G | NA | NA | 0.16111707841 | NA | NA | NA | S1 | RBD |
| 408 | R-S | 85.5504587156 | 88.4615384615 | 89.9838882922 | NA | NA | 33.3333333333 | S1 | RBD |
| 409 | Q-E | NA | NA | 0.107411385607 | NA | NA | NA | S1 | RBD |
| 411 | A-S | NA | NA | NA | 0.552486187845 | NA | NA | S1 | RBD |
| 415 | T-I | NA | NA | 0.0268528464017 | NA | NA | NA | S1 | RBD |
| 417 | K-N | 87.6146788991 | 90.8653846154 | 89.8496240602 | 0.552486187845 | NA | 100 | S1 | RBD |
| 418 | I-V | NA | NA | 0.0537056928034 | NA | NA | NA | S1 | RBD |
| 420 | D-N | NA | NA | 0.0268528464017 | NA | NA | NA | S1 | RBD |
| 421 | Y-F | NA | NA | 0.0268528464017 | NA | NA | NA | S1 | RBD |
| 430 | T-I | NA | NA | 0.0268528464017 | NA | NA | NA | S1 | RBD |
| 433 | V-F | NA | NA | 0.0268528464017 | NA | NA | NA | S1 | RBD |
| 438 | S-T | NA | NA | 0.0268528464017 | NA | NA | NA | S1 | RBD |
| 440 | N-K | 91.7431192661 | 94.7115384615 | 94.522019334 | NA | NA | 100 | S1 | RBD |
| 444 | K-R | NA | NA | 1.20837808808 | NA | NA | NA | S1 | RBD |
| 445 | V-H | NA | 88.9423076923 | NA | NA | NA | NA | S1 | RBD |
| 445 | V-P | 91.2844036697 | 0.961538461538 | 94.2266380236 | NA | NA | NA | S1 | RBD |
| 445 | V-S | NA | NA | 0.134264232009 | NA | NA | NA | S1 | RBD |
| 446 | G-I | NA | NA | 0.0537056928034 | NA | NA | NA | S1 | RBD |
| 446 | G-S | 91.2844036697 | 89.9038461538 | 94.387755102 | NA | NA | 33.3333333333 | S1 | RBD |
| 446 | G-V | NA | NA | NA | 0.552486187845 | NA | NA | S1 | RBD |
| 448 | N-S | NA | NA | 0.0268528464017 | NA | NA | NA | S1 | RBD |
| 450 | N-D | NA | 88.4615384615 | NA | NA | NA | NA | S1 | RBD |
| 452 | L-R | NA | NA | 0.0268528464017 | 100 | NA | NA | S1 | RBD |
| 452 | L-W | NA | 86.0576923077 | NA | NA | NA | NA | S1 | RBD |
| 455 | L-F | 0.917431192661 | NA | 0.107411385607 | NA | NA | NA | S1 | RBD |
| 455 | L-S | NA | 1.44230769231 | NA | NA | NA | NA | S1 | RBD |
| 455 | L-V | NA | NA | 0.0268528464017 | NA | NA | NA | S1 | RBD |
| 456 | F-L | 70.4128440367 | NA | 1.66487647691 | NA | NA | NA | S1 | RBD |
| 459 | S-F | NA | NA | NA | 0.552486187845 | NA | NA | S1 | RBD |
| 460 | N-K | 71.7889908257 | 90.3846153846 | 71.9119226638 | NA | NA | NA | S1 | RBD |
| 463 | P-L | 0.229357798165 | NA | NA | NA | NA | NA | S1 | RBD |
| 466 | R-K | NA | NA | 0.0268528464017 | NA | NA | NA | S1 | RBD |
| 471 | E-Q | NA | NA | 0.0268528464017 | NA | NA | NA | S1 | RBD |
| 474 | Q-H | NA | NA | 0.0268528464017 | NA | NA | NA | S1 | RBD |
| 474 | Q-K | NA | NA | 0.0805585392052 | NA | NA | NA | S1 | RBD |
| 476 | G-S | NA | NA | 0.0805585392052 | NA | NA | NA | S1 | RBD |
| 477 | S-D | NA | 0.961538461538 | 0.0268528464017 | NA | NA | NA | S1 | RBD |
| 477 | S-N | 75.4587155963 | 91.8269230769 | 73.8184747583 | NA | NA | 100 | S1 | RBD |
| 478 | T-E | NA | NA | 0.0268528464017 | NA | NA | NA | S1 | RBD |
| 478 | T-K | 75.6880733945 | 91.3461538462 | 70.3544575725 | 100 | NA | 100 | S1 | RBD |
| 478 | T-Q | NA | NA | 0.0268528464017 | NA | NA | NA | S1 | RBD |
| 478 | T-R | 0.45871559633 | 0.961538461538 | 3.59828141783 | NA | NA | NA | S1 | RBD |
| 481 | N-K | 0.229357798165 | 89.4230769231 | 0.0805585392052 | NA | NA | NA | S1 | RBD |
| 481 | N-S | NA | NA | 0.0268528464017 | NA | NA | NA | S1 | RBD |
| 483 | V-A | NA | NA | 0.0268528464017 | NA | NA | NA | S1 | RBD |
| 483 | V-K | NA | 0.961538461538 | NA | NA | NA | NA | S1 | RBD |
| 484 | E-A | 95.6422018349 | 1.44230769231 | 96.5628356606 | NA | NA | 100 | S1 | RBD |
| 484 | E-K | NA | 84.1346153846 | NA | NA | NA | NA | S1 | RBD |
| 484 | E-T | NA | NA | 0.0805585392052 | NA | NA | NA | S1 | RBD |
| 485 | G-D | NA | NA | 0.0537056928034 | NA | NA | NA | S1 | RBD |
| 485 | G-V | NA | NA | 0.0268528464017 | NA | NA | NA | S1 | RBD |
| 486 | F-H | NA | NA | 0.0268528464017 | NA | NA | NA | S1 | RBD |
| 486 | F-L | NA | NA | 0.0537056928034 | NA | NA | NA | S1 | RBD |
| 486 | F-P | 97.247706422 | 93.75 | 96.8850698174 | NA | NA | NA | S1 | RBD |
| 486 | F-S | NA | NA | 0.0268528464017 | NA | NA | NA | S1 | RBD |
| 486 | F-V | NA | NA | 0.0537056928034 | NA | NA | NA | S1 | RBD |
| 489 | Y-H | NA | 0.480769230769 | NA | NA | NA | NA | S1 | RBD |
| 490 | F-P | NA | NA | 0.0537056928034 | NA | NA | NA | S1 | RBD |
| 490 | F-S | 98.3944954128 | 0.480769230769 | 97.126745435 | NA | NA | NA | S1 | RBD |
| 492 | L-S | NA | NA | 0.0268528464017 | NA | NA | NA | S1 | RBD |
| 493 | Q-L | NA | NA | 0.0268528464017 | NA | NA | NA | S1 | RBD |
| 493 | Q-R | NA | NA | NA | NA | NA | 100 | S1 | RBD |
| 494 | S-P | NA | NA | 0.322234156821 | 0.552486187845 | NA | NA | S1 | RBD |
| 496 | G-S | NA | NA | NA | NA | NA | 33.3333333333 | S1 | RBD |
| 498 | Q-R | 97.4770642202 | 95.6730769231 | 96.8850698174 | NA | NA | 100 | S1 | RBD |
| 499 | P-L | NA | NA | 0.0537056928034 | NA | NA | NA | S1 | RBD |
| 501 | N-S | NA | NA | NA | 0.552486187845 | NA | NA | S1 | RBD |
| 501 | N-Y | 97.4770642202 | 95.6730769231 | 96.9119226638 | 0.552486187845 | 100 | 100 | S1 | RBD |
| 503 | V-F | 0.229357798165 | NA | NA | NA | NA | NA | S1 | RBD |
| 505 | Y-H | 97.0183486239 | 97.5961538462 | 96.723952739 | NA | NA | 100 | S1 | RBD |
| 510 | V-I | NA | NA | 0.0268528464017 | NA | NA | NA | S1 | RBD |
| 510 | V-L | NA | NA | 0.0537056928034 | NA | NA | NA | S1 | RBD |
| 511 | V-I | NA | NA | 0.107411385607 | NA | NA | NA | S1 | RBD |
| 514 | S-F | NA | NA | 0.0537056928034 | NA | NA | NA | S1 | RBD |
| 516 | E-Q | NA | NA | NA | 0.552486187845 | NA | NA | S1 | RBD |
| 517 | L-F | NA | NA | 0.0537056928034 | NA | NA | NA | S1 | RBD |
| 517 | L-P | NA | NA | 0.0268528464017 | NA | NA | NA | S1 | RBD |
| 521 | P-S | NA | NA | 2.68528464017 | NA | NA | NA | S1 | RBD |
| 521 | P-T | NA | NA | 0.187969924812 | NA | NA | NA | S1 | RBD |
| 522 | A-P | 0.229357798165 | NA | 0.0268528464017 | NA | NA | NA | S1 | RBD |
| 522 | A-V | 0.229357798165 | NA | 0.0268528464017 | NA | NA | NA | S1 | RBD |
| 524 | V-A | NA | NA | 0.0268528464017 | NA | NA | NA | S1 | RBD |
| 536 | N-K | NA | NA | 0.0805585392052 | NA | NA | NA | S1 | RBD |
| 540 | N-K | NA | NA | 0.0805585392052 | NA | NA | NA | S1 | RBD |
| 540 | N-Y | NA | NA | 0.0805585392052 | NA | NA | NA | S1 | RBD |
| 547 | T-I | NA | NA | 0.375939849624 | NA | NA | NA | S1 |  |
| 547 | T-K | 0.688073394495 | NA | NA | NA | NA | 33.3333333333 | S1 |  |
| 552 | L-F | NA | NA | 0.0268528464017 | NA | NA | NA | S1 |  |
| 554 | E-A | NA | NA | 0.349087003222 | NA | NA | NA | S1 |  |
| 554 | E-D | NA | NA | 0.0805585392052 | NA | NA | NA | S1 |  |
| 554 | E-K | NA | 99.5192307692 | 0.134264232009 | NA | NA | NA | S1 |  |
| 554 | E-Q | NA | NA | 0.107411385607 | NA | NA | NA | S1 |  |
| 558 | K-N | NA | NA | 0.0268528464017 | NA | NA | NA | S1 |  |
| 561 | P-H | NA | NA | 0.0268528464017 | NA | NA | NA | S1 |  |
| 561 | P-S | NA | NA | 0.0805585392052 | NA | NA | NA | S1 |  |
| 565 | F-L | 0.45871559633 | NA | 0.0268528464017 | NA | NA | NA | S1 |  |
| 570 | A-D | NA | NA | NA | NA | 100 | NA | S1 |  |
| 570 | A-G | NA | NA | 0.0268528464017 | NA | NA | NA | S1 |  |
| 570 | A-T | NA | NA | 0.0268528464017 | NA | NA | NA | S1 |  |
| 570 | A-V | NA | 99.5192307692 | NA | NA | NA | NA | S1 |  |
| 572 | T-I | NA | 0.480769230769 | 0.0537056928034 | 1.65745856354 | NA | NA | S1 |  |
| 573 | T-I | NA | NA | 0.456498388829 | NA | NA | NA | S1 |  |
| 574 | D-Y | NA | NA | 0.0268528464017 | NA | NA | NA | S1 |  |
| 580 | Q-L | NA | NA | 0.0268528464017 | NA | NA | NA | S1 |  |
| 582 | L-F | NA | NA | 0.0805585392052 | NA | NA | NA | S1 |  |
| 583 | E-D | NA | NA | 0.107411385607 | 0.552486187845 | 12.5 | NA | S1 |  |
| 583 | E-Q | NA | NA | 0.107411385607 | NA | NA | NA | S1 |  |
| 588 | T-A | NA | NA | 0.0268528464017 | NA | NA | NA | S1 |  |
| 588 | T-I | NA | NA | 0.107411385607 | NA | NA | NA | S1 |  |
| 594 | G-S | NA | 2.88461538462 | NA | NA | NA | NA | S1 |  |
| 607 | Q-H | NA | NA | NA | 0.552486187845 | NA | NA | S1 |  |
| 613 | Q-H | 0.229357798165 | NA | 0.241675617615 | 1.10497237569 | NA | NA | S1 |  |
| 614 | D-G | 98.8532110092 | 100 | 99.4092373792 | 100 | 100 | 100 | S1 |  |
| 621 | P-H | NA | NA | 0.0268528464017 | NA | NA | NA | S1 |  |
| 621 | P-L | NA | NA | 0.0268528464017 | NA | NA | NA | S1 |  |
| 621 | P-S | NA | 100 | 0.107411385607 | NA | NA | NA | S1 |  |
| 622 | V-I | NA | NA | 0.0268528464017 | NA | NA | NA | S1 |  |
| 622 | V-L | NA | NA | 0.0537056928034 | NA | NA | NA | S1 |  |
| 623 | A-S | NA | NA | 0.0537056928034 | NA | NA | NA | S1 |  |
| 623 | A-V | NA | NA | 0.0268528464017 | NA | NA | NA | S1 |  |
| 624 | I-M | NA | NA | 0.0268528464017 | NA | NA | NA | S1 |  |
| 628 | Q-R | NA | 0.480769230769 | NA | NA | NA | NA | S1 |  |
| 634 | R-H | NA | NA | 0.0268528464017 | NA | NA | NA | S1 |  |
| 635 | V-I | NA | NA | 0.0537056928034 | NA | NA | NA | S1 |  |
| 638 | T-I | NA | NA | 0.187969924812 | NA | NA | NA | S1 |  |
| 639 | G-A | NA | NA | 0.0268528464017 | NA | NA | NA | S1 |  |
| 640 | S-F | NA | NA | 0.16111707841 | NA | NA | NA | S1 |  |
| 641 | N-K | 0.229357798165 | NA | 0.0268528464017 | NA | NA | NA | S1 |  |
| 641 | N-Y | NA | NA | 0.0268528464017 | NA | NA | NA | S1 |  |
| 642 | V-F | NA | NA | 0.0268528464017 | NA | NA | NA | S1 |  |
| 642 | V-G | NA | NA | 0.0268528464017 | NA | NA | NA | S1 |  |
| 647 | A-S | NA | NA | 0.0268528464017 | NA | NA | NA | S1 |  |
| 654 | E-D | NA | NA | 0.0268528464017 | NA | NA | NA | S1 |  |
| 655 | H-Y | 99.7706422018 | 99.5192307692 | 99.7583243824 | NA | NA | 100 | S1 |  |
| 657 | N-T | NA | NA | 0.214822771214 | NA | NA | NA | S1 |  |
| 658 | N-S | NA | NA | 0.0537056928034 | NA | NA | NA | S1 |  |
| 659 | S-L | NA | NA | 0.107411385607 | NA | NA | NA | S1 |  |
| 660 | Y-F | NA | NA | 0.0805585392052 | NA | NA | NA | S1 |  |
| 661 | E-D | NA | NA | NA | 0.552486187845 | NA | NA | S1 |  |
| 666 | I-V | NA | NA | 1.02040816327 | NA | NA | NA | S1 |  |
| 668 | A-V | NA | NA | 0.0268528464017 | NA | NA | NA | S1 |  |
| 670 | I-V | NA | 0.961538461538 | 0.0268528464017 | NA | NA | NA | S1 |  |
| 675 | Q-H | NA | NA | 1.07411385607 | 0.552486187845 | NA | NA | S1 |  |
| 675 | Q-K | NA | NA | 0.0537056928034 | 0.552486187845 | NA | NA | S1 |  |
| 675 | Q-R | NA | NA | 0.0537056928034 | NA | NA | NA | S1 |  |
| 677 | Q-H | NA | NA | 0.0268528464017 | 1.10497237569 | NA | NA | S1 |  |
| 678 | T-I | NA | 0.480769230769 | 0.0268528464017 | 0.552486187845 | NA | NA | S1 |  |
| 679 | N-K | 97.9357798165 | 99.5192307692 | 99.5703544576 | NA | NA | 100 | S1 |  |
| 679 | N-M | NA | NA | 0.0268528464017 | NA | NA | NA | S1 |  |
| 679 | N-R | 0.917431192661 | NA | NA | NA | NA | NA | S1 |  |
| 680 | S-F | NA | NA | NA | 1.65745856354 | NA | NA | S1 |  |
| 680 | S-P | NA | NA | 0.0268528464017 | NA | NA | NA | S1 |  |
| 681 | P-H | 98.8532110092 | NA | 99.7851772288 | NA | 100 | 100 | S1 |  |
| 681 | P-R | NA | 99.5192307692 | NA | 100 | NA | NA | S1 |  |
| 683 | R-L | NA | NA | 0.0268528464017 | NA | NA | NA | S1 |  |
| 683 | R-W | NA | NA | 0.375939849624 | NA | NA | NA | S1 |  |
| 684 | A-T | NA | NA | 0.0805585392052 | NA | NA | NA | S1 |  |
| 684 | A-V | 0.229357798165 | NA | 0.0537056928034 | NA | NA | NA | S1 |  |
| 687 | V-L | NA | 0.480769230769 | NA | NA | NA | NA | S2 |  |
| 688 | A-V | NA | NA | 0.510204081633 | NA | NA | NA | S2 |  |
| 691 | S-F | NA | NA | 0.0268528464017 | NA | NA | NA | S2 |  |
| 692 | I-F | NA | NA | 0.0537056928034 | NA | NA | NA | S2 |  |
| 694 | A-V | NA | NA | NA | 0.552486187845 | NA | NA | S2 |  |
| 697 | M-I | NA | NA | 0.0268528464017 | NA | NA | NA | S2 |  |
| 698 | S-L | NA | NA | NA | 0.552486187845 | NA | NA | S2 |  |
| 701 | A-S | NA | NA | 0.0268528464017 | NA | NA | NA | S2 |  |
| 701 | A-V | NA | NA | 0.0805585392052 | NA | NA | NA | S2 |  |
| 702 | E-V | NA | NA | NA | 0.552486187845 | NA | NA | S2 |  |
| 703 | N-S | NA | NA | 0.0805585392052 | NA | NA | NA | S2 |  |
| 704 | S-L | 2.75229357798 | NA | 0.0805585392052 | NA | NA | NA | S2 |  |
| 708 | S-F | NA | NA | 0.0268528464017 | NA | NA | NA | S2 |  |
| 714 | I-L | 0.45871559633 | NA | NA | NA | NA | NA | S2 |  |
| 714 | I-V | NA | NA | 0.0268528464017 | NA | NA | NA | S2 |  |
| 716 | T-I | NA | NA | 0.0805585392052 | NA | 100 | NA | S2 |  |
| 719 | T-I | NA | NA | NA | 1.10497237569 | NA | NA | S2 |  |
| 731 | M-I | NA | NA | 0.0268528464017 | NA | NA | NA | S2 |  |
| 732 | T-I | 0.45871559633 | NA | 0.0537056928034 | NA | NA | NA | S2 |  |
| 735 | S-L | 0.229357798165 | NA | NA | NA | NA | NA | S2 |  |
| 742 | I-V | NA | NA | 0.16111707841 | NA | NA | NA | S2 |  |
| 747 | T-I | NA | NA | 0.0268528464017 | NA | NA | NA | S2 |  |
| 747 | T-N | NA | NA | 0.0268528464017 | NA | NA | NA | S2 |  |
| 748 | E-Q | NA | 0.961538461538 | NA | NA | NA | NA | S2 |  |
| 748 | E-V | NA | NA | 0.295381310419 | NA | NA | NA | S2 |  |
| 750 | S-I | NA | NA | 0.0268528464017 | NA | NA | NA | S2 |  |
| 752 | L-F | 0.229357798165 | NA | 0.0268528464017 | NA | NA | NA | S2 |  |
| 754 | L-S | NA | NA | 0.0268528464017 | NA | NA | NA | S2 |  |
| 755 | Q-H | NA | NA | 0.349087003222 | NA | NA | NA | S2 |  |
| 756 | Y-N | NA | NA | 0.0268528464017 | NA | NA | NA | S2 |  |
| 764 | N-I | NA | NA | 0.0268528464017 | NA | NA | NA | S2 |  |
| 764 | N-K | 98.8532110092 | 99.0384615385 | 99.0601503759 | NA | NA | 100 | S2 |  |
| 764 | N-R | NA | 0.480769230769 | NA | NA | NA | NA | S2 |  |
| 765 | R-C | 0.229357798165 | NA | NA | NA | NA | NA | S2 |  |
| 768 | T-I | NA | NA | 0.0268528464017 | NA | NA | NA | S2 |  |
| 769 | G-A | NA | NA | 0.0268528464017 | NA | NA | NA | S2 |  |
| 770 | I-V | NA | NA | 0.0537056928034 | NA | NA | NA | S2 |  |
| 771 | A-S | NA | NA | 0.0537056928034 | NA | NA | NA | S2 |  |
| 791 | T-I | NA | NA | NA | 1.10497237569 | NA | NA | S2 | FP |
| 793 | P-S | NA | NA | 0.0268528464017 | NA | NA | NA | S2 | FP |
| 796 | D-H | NA | NA | 0.107411385607 | NA | NA | NA | S2 | FP |
| 796 | D-Y | 99.7706422018 | 96.6346153846 | 99.4897959184 | 0.552486187845 | NA | 100 | S2 | FP |
| 797 | F-L | NA | NA | NA | NA | NA | 33.3333333333 | S2 | FP |
| 798 | G-S | NA | NA | 0.0268528464017 | NA | NA | NA | S2 | FP |
| 809 | P-L | NA | NA | 0.0268528464017 | NA | NA | NA | S2 |  |
| 809 | P-R | NA | NA | 0.0268528464017 | NA | NA | NA | S2 |  |
| 809 | P-S | 0.688073394495 | NA | 0.134264232009 | 1.10497237569 | NA | NA | S2 |  |
| 812 | P-L | NA | NA | 0.0805585392052 | 0.552486187845 | NA | NA | S2 |  |
| 812 | P-T | NA | NA | 0.0537056928034 | NA | NA | NA | S2 |  |
| 822 | L-F | NA | NA | 0.134264232009 | NA | NA | NA | S2 |  |
| 830 | D-Y | NA | NA | 0.0268528464017 | NA | NA | NA | S2 |  |
| 831 | A-V | NA | NA | 0.0537056928034 | NA | NA | NA | S2 |  |
| 834 | I-T | NA | NA | 0.0268528464017 | 0.552486187845 | NA | NA | S2 |  |
| 834 | I-V | NA | NA | 0.0537056928034 | NA | NA | NA | S2 |  |
| 842 | G-S | NA | NA | 0.0268528464017 | NA | NA | NA | S2 |  |
| 843 | D-G | NA | NA | 0.0268528464017 | NA | NA | NA | S2 |  |
| 843 | D-N | NA | NA | 0.0268528464017 | NA | NA | NA | S2 |  |
| 844 | I-T | NA | NA | 0.0268528464017 | NA | NA | NA | S2 |  |
| 844 | I-V | NA | NA | 0.0268528464017 | NA | NA | NA | S2 |  |
| 846 | A-S | NA | NA | 0.214822771214 | NA | NA | NA | S2 |  |
| 850 | I-L | NA | NA | NA | 0.552486187845 | NA | NA | S2 |  |
| 852 | A-S | NA | NA | 0.0805585392052 | NA | NA | NA | S2 |  |
| 854 | K-N | NA | NA | 0.0268528464017 | NA | NA | NA | S2 |  |
| 854 | K-R | NA | NA | 0.0268528464017 | NA | NA | NA | S2 |  |
| 856 | N-K | NA | NA | NA | NA | NA | 33.3333333333 | S2 |  |
| 859 | T-I | NA | NA | NA | 0.552486187845 | NA | NA | S2 |  |
| 859 | T-N | NA | NA | NA | 0.552486187845 | NA | NA | S2 |  |
| 860 | V-L | NA | NA | 0.0268528464017 | NA | NA | NA | S2 |  |
| 867 | D-N | NA | NA | 0.0268528464017 | NA | NA | NA | S2 |  |
| 871 | A-S | NA | NA | NA | 0.552486187845 | NA | NA | S2 |  |
| 879 | A-S | NA | NA | 0.0268528464017 | NA | NA | NA | S2 |  |
| 879 | A-T | NA | NA | 0.0268528464017 | NA | NA | NA | S2 |  |
| 883 | T-I | NA | NA | 0.912996777658 | NA | NA | NA | S2 |  |
| 885 | G-D | NA | NA | 0.0268528464017 | NA | NA | NA | S2 |  |
| 890 | A-S | NA | NA | 0.0268528464017 | NA | NA | NA | S2 |  |
| 890 | A-V | NA | NA | 0.0268528464017 | NA | NA | NA | S2 |  |
| 899 | A-S | 0.229357798165 | NA | NA | NA | NA | NA | S2 |  |
| 915 | V-I | NA | NA | 0.0537056928034 | NA | NA | NA | S2 | HR1 |
| 922 | L-F | NA | 1.92307692308 | NA | NA | NA | NA | S2 | HR1 |
| 929 | S-T | NA | NA | NA | 0.552486187845 | NA | NA | S2 | HR1 |
| 932 | G-S | NA | NA | 0.0268528464017 | NA | NA | NA | S2 | HR1 |
| 938 | L-F | NA | NA | NA | 0.552486187845 | NA | NA | S2 | HR1 |
| 939 | S-F | NA | 99.5192307692 | 0.107411385607 | NA | NA | NA | S2 | HR1 |
| 940 | S-F | NA | NA | 0.0268528464017 | NA | NA | NA | S2 | HR1 |
| 942 | A-S | NA | NA | 0.0268528464017 | NA | NA | NA | S2 | HR1 |
| 943 | S-I | NA | NA | 0.0268528464017 | NA | NA | NA | S2 | HR1 |
| 950 | D-N | NA | NA | NA | 100 | NA | NA | S2 | HR1 |
| 952 | V-I | NA | NA | 0.214822771214 | NA | NA | NA | S2 | HR1 |
| 954 | Q-H | 100 | 99.5192307692 | 99.9194414608 | NA | NA | 100 | S2 | HR1 |
| 960 | N-T | NA | NA | 0.0268528464017 | NA | NA | NA | S2 | HR1 |
| 961 | T-M | NA | NA | 0.214822771214 | NA | NA | NA | S2 | HR1 |
| 964 | K-E | NA | NA | 1.87969924812 | NA | NA | NA | S2 | HR1 |
| 969 | N-K | 99.0825688073 | 100 | 99.8120300752 | NA | NA | 100 | S2 | HR1 |
| 972 | A-S | NA | NA | 0.0268528464017 | NA | NA | NA | S2 | HR1 |
| 978 | N-S | NA | NA | 0.268528464017 | NA | NA | NA | S2 | HR1 |
| 979 | D-E | NA | NA | NA | 0.552486187845 | NA | NA | S2 | HR1 |
| 981 | L-F | NA | NA | NA | NA | NA | 33.3333333333 | S2 | HR1 |
| 982 | S-A | NA | NA | NA | NA | 100 | NA | S2 | HR1 |
| 998 | T-A | NA | NA | 0.214822771214 | NA | NA | NA | S2 |  |
| 1003 | S-I | NA | NA | 0.0537056928034 | NA | NA | NA | S2 |  |
| 1008 | V-M | 0.229357798165 | NA | NA | NA | NA | NA | S2 |  |
| 1009 | T-I | NA | NA | 0.0268528464017 | NA | NA | NA | S2 |  |
| 1014 | R-T | NA | NA | 0.0268528464017 | NA | NA | NA | S2 |  |
| 1020 | A-G | NA | NA | 0.0268528464017 | NA | NA | NA | S2 |  |
| 1020 | A-S | NA | NA | 0.0268528464017 | NA | NA | NA | S2 |  |
| 1020 | A-T | 0.229357798165 | NA | NA | NA | NA | NA | S2 |  |
| 1020 | A-V | NA | NA | 0.187969924812 | NA | NA | NA | S2 |  |
| 1023 | N-I | 0.229357798165 | NA | NA | NA | NA | NA | S2 |  |
| 1023 | N-S | NA | NA | 0.0268528464017 | NA | NA | NA | S2 |  |
| 1041 | D-E | NA | NA | 0.0268528464017 | NA | NA | NA | S2 |  |
| 1045 | K-R | NA | NA | 1.10096670247 | NA | NA | NA | S2 |  |
| 1050 | M-L | NA | NA | 0.0537056928034 | NA | NA | NA | S2 |  |
| 1058 | H-R | NA | NA | 0.0805585392052 | NA | NA | NA | S2 |  |
| 1058 | H-Y | 0.45871559633 | NA | 0.0268528464017 | NA | NA | NA | S2 |  |
| 1070 | A-V | NA | NA | 0.0268528464017 | NA | NA | NA | S2 |  |
| 1071 | Q-K | NA | NA | 0.0268528464017 | NA | NA | NA | S2 |  |
| 1071 | Q-L | NA | NA | 0.0268528464017 | NA | NA | NA | S2 |  |
| 1072 | E-K | NA | NA | 0.0268528464017 | NA | NA | NA | S2 |  |
| 1073 | K-N | NA | NA | 0.0268528464017 | 0.552486187845 | NA | NA | S2 |  |
| 1073 | K-T | NA | NA | 0.0268528464017 | NA | NA | NA | S2 |  |
| 1079 | P-S | NA | NA | 0.0268528464017 | NA | NA | NA | S2 |  |
| 1084 | D-G | NA | NA | 0.375939849624 | NA | NA | NA | S2 |  |
| 1085 | G-R | NA | NA | 0.0805585392052 | NA | NA | NA | S2 |  |
| 1087 | A-S | 0.229357798165 | NA | 0.0805585392052 | NA | NA | NA | S2 |  |
| 1096 | V-I | NA | NA | 0.0268528464017 | NA | NA | NA | S2 |  |
| 1099 | G-D | NA | NA | 0.0537056928034 | NA | NA | NA | S2 |  |
| 1099 | G-S | NA | NA | 0.0268528464017 | NA | NA | NA | S2 |  |
| 1101 | H-Y | 0.229357798165 | NA | 0.0805585392052 | 1.10497237569 | NA | NA | S2 |  |
| 1104 | V-I | NA | NA | 0.107411385607 | NA | NA | NA | S2 |  |
| 1104 | V-L | NA | NA | NA | 1.65745856354 | NA | NA | S2 |  |
| 1117 | T-I | NA | NA | 0.241675617615 | NA | NA | NA | S2 |  |
| 1118 | D-H | NA | NA | NA | NA | 100 | NA | S2 |  |
| 1122 | V-A | NA | NA | 0.0268528464017 | NA | NA | NA | S2 |  |
| 1122 | V-L | 0.917431192661 | NA | 0.0268528464017 | NA | NA | NA | S2 |  |
| 1122 | V-M | NA | NA | 0.0805585392052 | NA | NA | NA | S2 |  |
| 1124 | G-V | NA | NA | 0.241675617615 | NA | NA | NA | S2 |  |
| 1127 | D-A | NA | NA | 0.0268528464017 | NA | NA | NA | S2 |  |
| 1128 | V-L | 0.688073394495 | NA | 0.16111707841 | NA | NA | NA | S2 |  |
| 1133 | V-F | NA | NA | 0.0268528464017 | NA | NA | NA | S2 |  |
| 1133 | V-I | NA | NA | 0.0268528464017 | NA | NA | NA | S2 |  |
| 1139 | D-B | NA | 0.480769230769 | NA | NA | NA | NA | S2 |  |
| 1139 | D-H | NA | NA | 0.0268528464017 | NA | NA | NA | S2 |  |
| 1139 | D-N | 0.229357798165 | 2.40384615385 | NA | NA | NA | NA | S2 |  |
| 1143 | P-L | NA | 100 | 0.0537056928034 | NA | NA | NA | S2 |  |
| 1143 | P-S | NA | NA | 0.0268528464017 | NA | NA | NA | S2 |  |
| 1146 | D-H | NA | NA | 0.375939849624 | NA | NA | NA | S2 |  |
| 1146 | D-N | NA | NA | 2.47046186896 | NA | NA | NA | S2 |  |
| 1146 | D-V | NA | NA | 0.0268528464017 | NA | NA | NA | S2 |  |
| 1150 | E-V | NA | NA | 0.0268528464017 | NA | NA | NA | S2 |  |
| 1153 | D-Y | NA | NA | 0.0268528464017 | 0.552486187845 | NA | NA | S2 |  |
| 1157 | K-N | NA | NA | 0.0268528464017 | NA | NA | NA | S2 |  |
| 1159 | H-L | NA | NA | 0.0268528464017 | NA | NA | NA | S2 |  |
| 1159 | H-R | NA | NA | 0.0268528464017 | NA | NA | NA | S2 |  |
| 1162 | P-L | NA | NA | 0.0805585392052 | NA | NA | NA | S2 |  |
| 1162 | P-S | NA | NA | 0.0805585392052 | 0.552486187845 | NA | NA | S2 |  |
| 1163 | D-Y | NA | NA | 0.0268528464017 | NA | NA | NA | S2 | HR2 |
| 1165 | D-N | NA | NA | 0.0805585392052 | NA | NA | NA | S2 | HR2 |
| 1167 | G-V | NA | NA | NA | 0.552486187845 | NA | NA | S2 | HR2 |
| 1176 | V-F | NA | NA | 0.0537056928034 | NA | NA | NA | S2 | HR2 |
| 1177 | V-L | NA | NA | 0.0268528464017 | NA | NA | NA | S2 | HR2 |
| 1178 | N-D | 0.45871559633 | NA | NA | NA | NA | NA | S2 | HR2 |
| 1181 | K-I | NA | NA | 0.0805585392052 | NA | NA | NA | S2 | HR2 |
| 1182 | E-Q | NA | NA | 0.0268528464017 | NA | NA | NA | S2 | HR2 |
| 1183 | I-V | NA | NA | 0.0268528464017 | NA | NA | NA | S2 | HR2 |
| 1184 | D-Y | NA | NA | 0.0268528464017 | NA | NA | NA | S2 | HR2 |
| 1186 | L-F | NA | NA | 0.0268528464017 | NA | NA | NA | S2 | HR2 |
| 1188 | E-A | NA | NA | 0.0268528464017 | NA | NA | NA | S2 | HR2 |
| 1191 | K-N | NA | NA | NA | 0.552486187845 | 12.5 | NA | S2 | HR2 |
| 1199 | D-N | NA | NA | NA | 0.552486187845 | NA | NA | S2 | HR2 |
| 1201 | Q-H | NA | NA | 0.0268528464017 | NA | NA | NA | S2 | HR2 |
| 1201 | Q-K | NA | NA | 0.0268528464017 | NA | NA | NA | S2 | HR2 |
| 1202 | E-Q | 0.229357798165 | NA | NA | NA | NA | NA | S2 | HR2 |
| 1202 | E-V | NA | NA | 0.0537056928034 | NA | NA | NA | S2 | HR2 |
| 1203 | L-F | NA | NA | 0.0268528464017 | NA | NA | NA | S2 | HR2 |
| 1208 | Q-H | NA | NA | 0.0268528464017 | NA | NA | NA | S2 | HR2 |
| 1208 | Q-R | NA | NA | 0.0268528464017 | NA | NA | NA | S2 | HR2 |
| 1210 | I-V | 0.688073394495 | NA | NA | NA | NA | NA | S2 | HR2 |
| 1216 | I-V | NA | NA | 0.0268528464017 | NA | NA | NA | S2 | TM |
| 1218 | L-I | NA | NA | 0.0268528464017 | NA | NA | NA | S2 | TM |
| 1219 | G-V | NA | NA | 0.0268528464017 | 0.552486187845 | NA | NA | S2 | TM |
| 1228 | V-A | 0.229357798165 | NA | 0.0537056928034 | NA | NA | NA | S2 | TM |
| 1228 | V-L | NA | NA | 0.0805585392052 | NA | NA | NA | S2 | TM |
| 1229 | M-I | NA | 0.961538461538 | NA | NA | NA | NA | S2 | TM |
| 1233 | M-V | NA | NA | 0.0268528464017 | NA | NA | NA | S2 | TM |
| 1234 | L-I | NA | NA | 0.0268528464017 | 0.552486187845 | NA | NA | S2 | TM |
| 1235 | C-F | 0.229357798165 | NA | NA | NA | NA | NA | S2 | TM |
| 1237 | M-I | 0.229357798165 | NA | NA | 0.552486187845 | NA | NA | S2 | TM |
| 1237 | M-V | NA | NA | 0.0268528464017 | NA | NA | NA | S2 | TM |
| 1238 | T-I | NA | NA | 0.0268528464017 | NA | NA | NA | S2 | CT |
| 1243 | C-R | NA | NA | 0.0537056928034 | NA | NA | NA | S2 | CT |
| 1247 | C-F | NA | NA | 0.0537056928034 | NA | NA | NA | S2 | CT |
| 1247 | C-S | NA | NA | 0.0268528464017 | NA | NA | NA | S2 | CT |
| 1248 | C-Y | 0.229357798165 | NA | NA | NA | NA | NA | S2 | CT |
| 1249 | S-A | NA | NA | 0.0268528464017 | NA | NA | NA | S2 | CT |
| 1249 | S-P | NA | 0.480769230769 | NA | NA | NA | NA | S2 | CT |
| 1250 | C-F | NA | NA | 0.0268528464017 | NA | NA | NA | S2 | CT |
| 1251 | G-V | NA | NA | 0.0268528464017 | NA | NA | NA | S2 | CT |
| 1254 | C-S | NA | NA | 0.0268528464017 | NA | NA | NA | S2 | CT |
| 1255 | K-R | NA | NA | 0.993555316864 | NA | NA | NA | S2 | CT |
| 1259 | D-N | 0.229357798165 | NA | NA | NA | NA | NA | S2 | CT |
| 1259 | D-Y | NA | NA | NA | 0.552486187845 | NA | NA | S2 | CT |
| 1260 | D-H | NA | NA | 0.0268528464017 | NA | NA | NA | S2 | CT |
| 1260 | D-Y | NA | NA | NA | 0.552486187845 | NA | NA | S2 | CT |
| 1263 | P-L | NA | NA | 0.0537056928034 | 0.552486187845 | NA | NA | S2 | CT |
| 1264 | V-L | 0.229357798165 | NA | 0.268528464017 | 3.86740331492 | NA | NA | S2 | CT |

SP: signal peptide (amino acids residues: 1-13), NTD: N-terminal subdomain (14-305 residues),

RBD: C-terminal receptor binding subdomain (319-541 residues),

FP: fusion peptide (788–806 residues), HR1: heptapeptide repeat sequence 1 (912–984 residues),

HR2: heptapeptide repeat sequence 2 (1163–1213 residues), CT: C-terminal tail (1237–1273).
