# Supplementary material for: Investigating the evolutionary dynamics and mutational pattern of SARS-CoV-2 spike gene on selected SARS-CoV-2 variants
Source: PLoS One. 2025 Oct 21;20(10):e0333093. doi: 10.1371/journal.pone.0333093 (PMC12539718; doi:10.1371/journal.pone.0333093)
Supplement: S2 Fig — The figure shows frequencies higher than 10% in at least one of the analyzed variants. Nearly 85% (77 out of 91) of these high-frequency mutations are found in the S1 subunit, distributed as follows: 29 in the NTD subdomain, 37 in the RBD, and 11 in the region between S1 and S2. Within the S2 subunit, most mutations are concentrated in the HR1 subdomain, with a single mutation observed in the fusion peptide (FP) and another in HR2. Crucially, the N501Y and D614G substitutions are present in all variants, while A27S and G142D are common to five variants, excluding Alpha. S1: Subunit 1 (14–685 residues): NTD: N-terminal subdomain (14–305 residues), RBD: C-terminal receptor binding subdomain (319–541 residues). S2: Subunit 2 (686–1273 residues): FP: Fusion peptide (788–806 residues), HR1: Heptapeptide repeat sequence 1 (912–984 residues), HR2: Heptapeptide repeat sequence 2 (1163–1213 residues). (DOCX) [file pone.0333093.s005.docx]

**
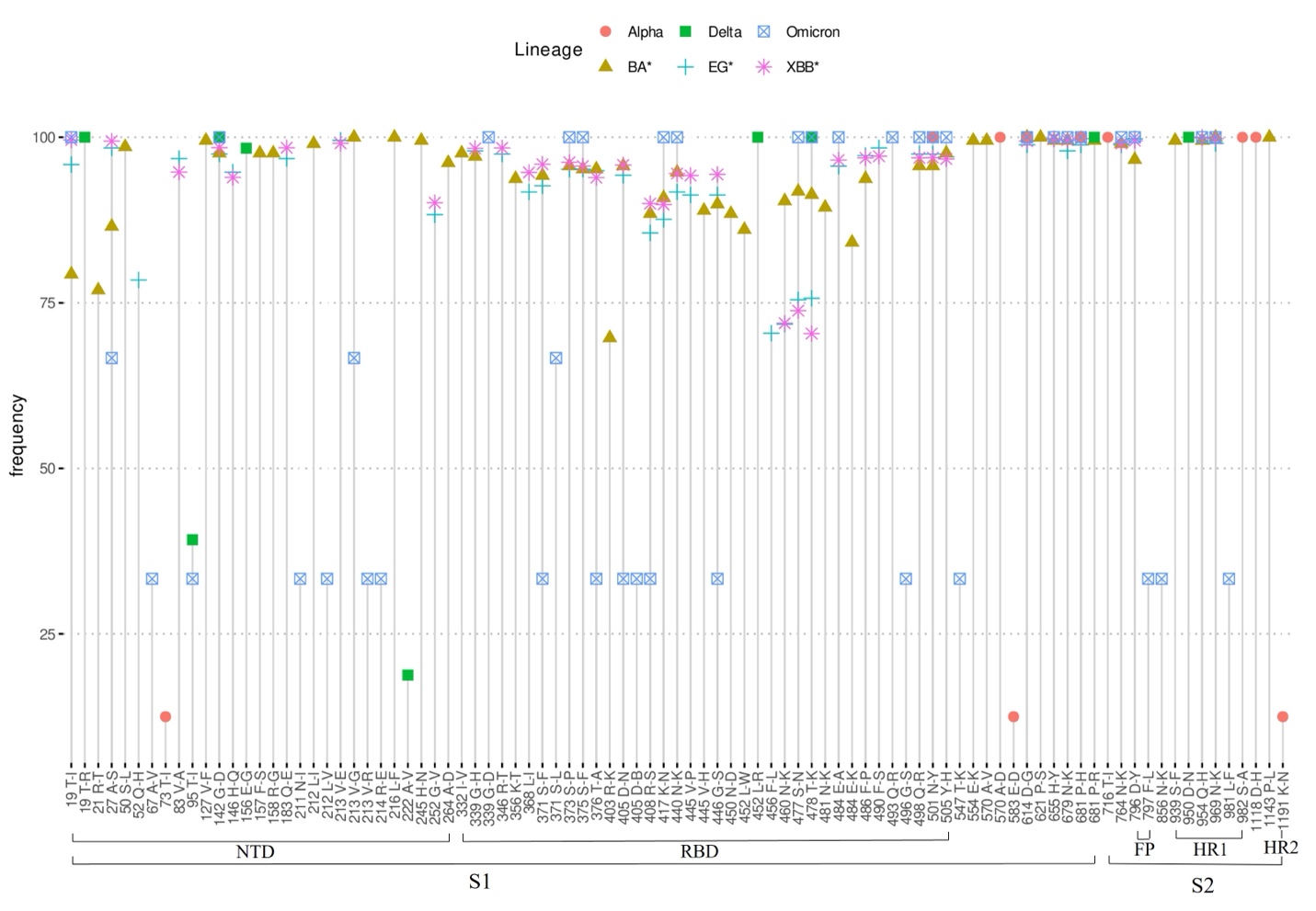
Supplementary Figure 2.** Highly frequent (>10%) Spike gene amino acid substitutions sites. Substitutions frequency higher than 10% in at least one of the analyzed variants is shown. Almost 85% (77 out of 91) of mutations are found in the S1 subunit: 29 in the NTD subdomain, 37 in the RBD and 11 between S1/S2 (between RBD and FP). In the S2 subunit, most mutations are in the HR1 subdomain (n=6), followed by two mutations in the fusion peptide (FP) and one in HR2. Notably, N501Y and D614G substitutions are present in all variants, while A27S and G142D are common to five variants, except Alpha.

S1: Subunit 1 (14-685 residues): NTD: N-terminal subdomain (14-305 residues), RBD: C-terminal receptor binding subdomain (319-541 residues).

S2: Subunit 2 (686-1273 residues): FP: Fusion peptide (788-806 residues), HR1: Heptapeptide repeat sequence 1 (912-984 residues), HR2: Heptapeptide repeat sequence 2 (1163-1213 residues).
