# Supplementary material for: Investigating the evolutionary dynamics and mutational pattern of SARS-CoV-2 spike gene on selected SARS-CoV-2 variants
Source: PLoS One. 2025 Oct 21;20(10):e0333093. doi: 10.1371/journal.pone.0333093 (PMC12539718; doi:10.1371/journal.pone.0333093)
Supplement: S4 Table — Each deletion was attributed to the functional Spike subunits and the corresponding subdomain. Frequencies are reported in percentage scale. (DOCX) [file pone.0333093.s006.docx]

**Table S4.** Amino acid deletions frequencies of Spike protein coding gene of Alpha, Delta, Omicron, XBB*, EG*, BA* variants with Wuhan-Hu-1 (NC_045512.2) as reference.
Each deletion was attributed to the functional spike subunits and the corresponding subdomain.
Frequencies are reported in percentage scale.

| **Site** | **Deletion** | **Alpha-Frequency** | **Delta-Frequency** | **Omicron-Frequency** | **XBB*-Frequency** | **EG*-Frequency** | **AB*-Frequency** | **Subunits** | **Subunits** |
| --- | --- | --- | --- | --- | --- | --- | --- | --- | --- |
| 13 | SQC | NA | NA | NA | 0.0268528464017 | NA | NA | S1 | SP |
| 14 | QCV | NA | NA | NA | 0.0268528464017 | NA | NA | S1 | NTD |
| 16 | V | NA | NA | NA | 0.0268528464017 | NA | NA | S1 | NTD |
| 24 | LPP | NA | NA | 66.6666666667 | 99.1944146079 | 98.3944954128 | 70.6730769231 | S1 | NTD |
| 25 | PPA | NA | NA | NA | 0.0537056928034 | NA | NA | S1 | NTD |
| 69 | HV | 100 | NA | 33.3333333333 | 0.107411385607 | NA | 85.5769230769 | S1 | NTD |
| 85 | PFNDG | NA | NA | NA | 0.0268528464017 | NA | NA | S1 | NTD |
| 139 | PFLGVYYH | NA | NA | NA | 0.0537056928034 | NA | NA | S1 | NTD |
| 140 | FLGVY | NA | NA | NA | 0.0268528464017 | NA | NA | S1 | NTD |
| 141 | L | NA | NA | NA | 0.0268528464017 | NA | NA | S1 | NTD |
| 141 | LGVY | NA | NA | NA | 0.107411385607 | NA | NA | S1 | NTD |
| 141 | LGVYYH | NA | NA | NA | 0.0268528464017 | NA | NA | S1 | NTD |
| 143 | VY | NA | NA | NA | 0.0537056928034 | 0.229357798165 | NA | S1 | NTD |
| 143 | VYY | NA | NA | 33.3333333333 | NA | NA | NA | S1 | NTD |
| 144 | Y | 100 | NA | NA | 0.0268528464017 | NA | 94.7115384615 | S1 | NTD |
| 145 | Y | NA | NA | NA | 98.6573576799 | 98.3944954128 | NA | S1 | NTD |
| 145 | YH | NA | NA | NA | 0.0268528464017 | NA | NA | S1 | NTD |
| 156 | EF | NA | 1.10497237569 | NA | NA | NA | NA | S1 | NTD |
| 157 | FR | NA | 97.2375690608 | NA | NA | NA | NA | S1 | NTD |
| 175 | FLM | NA | NA | NA | 0.0268528464017 | NA | NA | S1 | NTD |
| 185 | NFKN | NA | NA | NA | 0.0537056928034 | NA | NA | S1 | NTD |
| 186 | FKN | NA | NA | NA | 0.0268528464017 | NA | NA | S1 | NTD |
| 210 | I | NA | NA | NA | 0.0537056928034 | 0.229357798165 | NA | S1 | NTD |
| 211 | N | NA | NA | NA | NA | NA | 99.0384615385 | S1 | NTD |
| 241 | LLA | NA | NA | NA | 0.16111707841 | NA | NA | S1 | NTD |
| 242 | LA | NA | NA | NA | 0.107411385607 | NA | NA | S1 | NTD |
| 245 | H | NA | NA | NA | 0.0268528464017 | NA | NA | S1 | NTD |
| 371 | SAS | NA | NA | NA | NA | 0.229357798165 | NA | S1 | RBD |
| 375 | ST | NA | NA | NA | 0.0268528464017 | NA | NA | S1 | RBD |
| 483 | V | NA | NA | NA | NA | NA | 75 | S1 | RBD |
| 484 | E | NA | NA | NA | NA | NA | 0.961538461538 | S1 | RBD |
| 640 | S | NA | NA | NA | 0.0268528464017 | NA | NA | S1 |  |
| 1257 | D | NA | NA | NA | 0.0805585392052 | NA | NA | S2 | CT |

SP: signal peptide (amino acids residues: 1-13), NTD: N-terminal subdomain (14-305 residues),
CT: C-terminal tail (1237–1273).
